# Dynamic Metal-Coordinated Adhesive and Self-Healable Antifreezing Hydrogels for Strain Sensing, Flexible Supercapacitors, and EMI Shielding Applications

**DOI:** 10.1021/acsomega.4c04851

**Published:** 2024-07-21

**Authors:** Ashis Ghosh, Sudhir Kumar, Prem Pal Singh, Suvendu Nandi, Mahitosh Mandal, Debabrata Pradhan, Bhanu Bhusan Khatua, Rajat Kumar Das

**Affiliations:** † Materials Science Centre, Indian Institute of Technology Kharagpur, Kharagpur 721302, India; ‡ School of Medical Science and Technology, Indian Institute of Technology Kharagpur, Kharagpur 721302, India

## Abstract

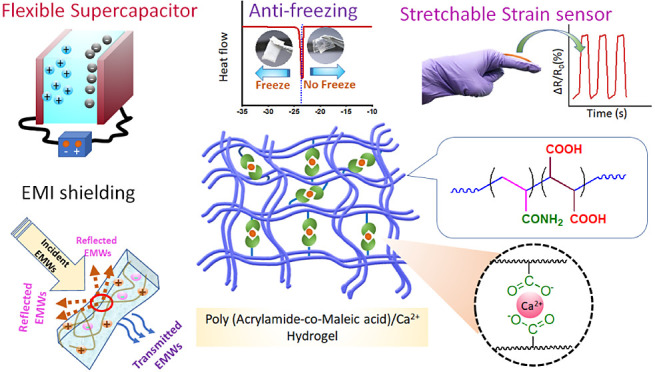

Dynamic metal-coordinated adhesive and self-healable hydrogel materials have garnered significant attention in recent years due to their potential applications in various fields. These hydrogels can form reversible metal–ligand bonds, resulting in a network structure that can be easily broken and reformed, leading to self-healing capabilities. In addition, these hydrogels possess excellent mechanical strength and flexibility, making them suitable for strain-sensing applications. In this work, we have developed a mechanically robust, highly stretchable, self-healing, and adhesive hydrogel by incorporating Ca^2+^-dicarboxylate dynamic metal–ligand cross-links in combination with low density chemical cross-links into a poly(acrylamide-*co*-maleic acid) copolymer structure. Utilizing the reversible nature of the Ca^2+^-dicarboxylate bond, the hydrogel exhibited a tensile strength of up to ∼250 kPa and was able to stretch to 15–16 times its original length. The hydrogel exhibited a high fracture energy of ∼1500 J m^–2^, similar to that of cartilage. Furthermore, the hydrogel showed good recovery, fatigue resistance, and fast self-healing properties due to the reversible Ca^2+^-dicarboxylate cross-links. The presence of Ca^2+^ resulted in a highly conductive hydrogel, which was utilized to design a flexible resistive strain sensor. This hydrogel can strongly adhere to different substrates, making it advantageous for applications in flexible electronic devices. When adhered to human body parts, the hydrogel can efficiently detect limb movements. The hydrogel also exhibited excellent performance as a solid electrolyte for flexible supercapacitors, with a capacitance of ∼260 F/g at 0.5 A/g current density. Due to its antifreezing and antidehydration properties, this hydrogel retains its flexibility at subzero temperatures for an extended period. Additionally, the porous network and high water content of the hydrogel impart remarkable electromagnetic attenuation properties, with a value of ∼38 dB in the 14.5–20.5 GHz frequency range, which is higher than any other hydrogel without conducting fillers. Overall, the hydrogel reported in this study exhibits diverse applications as a strain sensor, solid electrolyte for flexible supercapacitors, and efficient material for electromagnetic attenuation. Its multifunctional properties make it a promising candidate for use in various fields as a state-of-the-art material.

## Introduction

In recent years, hydrogels have found extensive applications in flexible electronics like soft robotics,^1^ human health monitoring,^2,3^ supercapacitor,^4^ biosensor,^5^ artificial skin^6−10^ because of their soft and wet nature, high flexibility, conductive nature, tunable stimuli-responsive functionality, ability to convert mechanical deformation into electrical signals, and structural similarity to biological tissues. However, hydrogel-based electronic materials often suffer from issues such as low mechanical strength and a lack of reversibility during repeated load-bearing applications, which can affect their durability and efficiency. Moreover, when exposed to air, the water content of hydrogels gradually decreases, causing a loss of their prime functional properties, such as stretchability, flexibility, and conductivity. Additionally, for conventional hydrogel materials, the water inside the hydrogel freezes at subzero temperatures, reducing their flexibility and conductivity, and limiting their applications at low temperatures. Consequently, for instance, flexible electronic materials based on such hydrogels become inefficient in colder climates. Therefore, there is a need to design a hydrogel that is not only mechanically robust and conductive but also self-healable, self-recoverable, and resistant to freezing and drying, to enable its multifunctional application. To enhance the mechanical robustness of hydrogels, various strategies have been explored, such as forming macromolecular microsphere hydrogels,^11^ slide ring hydrogels,^12^ DN hydrogels,^13−15^ and nanocomposite hydrogels.^16^ Among these, double network (DN) hydrogels have gained significant attention due to their unique design strategy. DN hydrogels consist of a highly cross-linked brittle first network and a lightly cross-linked flexible second network. When subjected to a load, the densely cross-linked network ruptures to dissipate the energy, while the flexible network maintains the structural integrity.^15^ This easily breakable network, known as the sacrificial network, can be made of either covalent or noncovalent bonds. Using noncovalent bonds as sacrificial bonds is more advantageous, as they have reversible properties, enabling better recoverability and self-healing of the material. Further advancements in DN systems led to the development of dual cross-linked hydrogels, where the polymer is cross-linked with two different types of cross-linkers. This results in one cross-linker providing structural integrity, while the other acts as the sacrificial bond. When the load is applied, the sacrificial bond breaks to dissipate energy, enhancing the toughness of the material. The reversibility of these sacrificial bonds allows the hydrogel to recover its dissipated energy once the load is removed, enabling self-healing and antifatigue properties. Various noncovalent bonds, such as hydrogen bonding interaction,^17^ metal–ligand interaction,^18^ ionic interaction,^19^ and hydrophobic interaction^20^ have been used to introduce self-healing property, good recoverability, antifatigue property inside the hydrogel. Among these, sacrificial bonds based on metal–ligand interactions have gained significant attention, as they provide a wide range of mechanical strength, stimuli-responsiveness, and conductivity, making hydrogels suitable for multifunctional and conductive applications. For instance, Holten-Andersen et al.^21^ developed a polymer hydrogel with a catechol ligand that increased its mechanical strength by coordinating with Fe^3+^ ions. The strength of the polymer hydrogel was influenced by the formation of mono-, bis-, and tris complexes of Fe^3+^ in varying pH environments. Guo and coworkers introduced dual dynamic cross-linking strategy where pH-responsive Fe^3+^–catechol interaction was combined with reversible Schiff base bonds^22^ or ureido-pyrimidone hydrogen bonding interaction^23^ to fabricate adhesive, biocompatible, self-healing, and injectable hydrogel materials for wound healing applications. In addition to the catechol ligand, other ligands such as carboxylate,^24^ terpyridine,^25^ and imidazole^26^ are commonly utilized in the development of coordination complex-based hydrogels and the mechanical strength of these materials also vary according to different metal–ligand combinations. Carboxylate ions can form complex with various bivalent and trivalent metal ions and the strength of the materials can differ significantly by changing the metal ions as well as the counterions.^27−30^ Zhang et al. showed that, when compared to other metal ions, carboxylate ions can bind more strongly with Fe^3+^ ions.^31^ Zhou and coworkers synthesized a poly(acrylamide-co maleic acid) hydrogel incorporating Fe^3+^-carboxylate-based cross-linking, achieving a tensile strength of ∼5.9 MPa and 8–9 times stretchability.^32^ Jeon and group introduced a staggered coordination structure of Fe^3+^ ions with a dicarboxylate ligand, enhancing the mechanical strength up to 12 MPa. These materials also demonstrated faster recovery properties.^33^ Although Fe^3+^-carboxylate-based cross-linking provides superior mechanical strength and toughness, the strechability, water content, and ionic conductivity of the resultant hydrogels decreased with increasing cross-linking density. These materials also lacked self-healing ability.^24^ In our previous study, we demonstrated the complex formation ability of dicarboxylate moieties with divalent or trivalent metal ions by soaking them in different metal ion solutions.^18^ Our work demonstrated that in the case of Fe^3+^ and Fe^2+^ ions, mechanical properties of the hydrogels increased drastically but these materials exhibited poor self-healing ability and low conductivity. The use of other bivalent metal ions like Ca^2+^, Cu^2+^, Zn^2+^, and Ni^2+^ can increase the conductivity and improve other functional properties such as fast self-healing, adhesion, and water content.^18^ But their mechanical properties did not show a significant increase because of swelling during soaking in the metal ion solution.

**Scheme 1:**
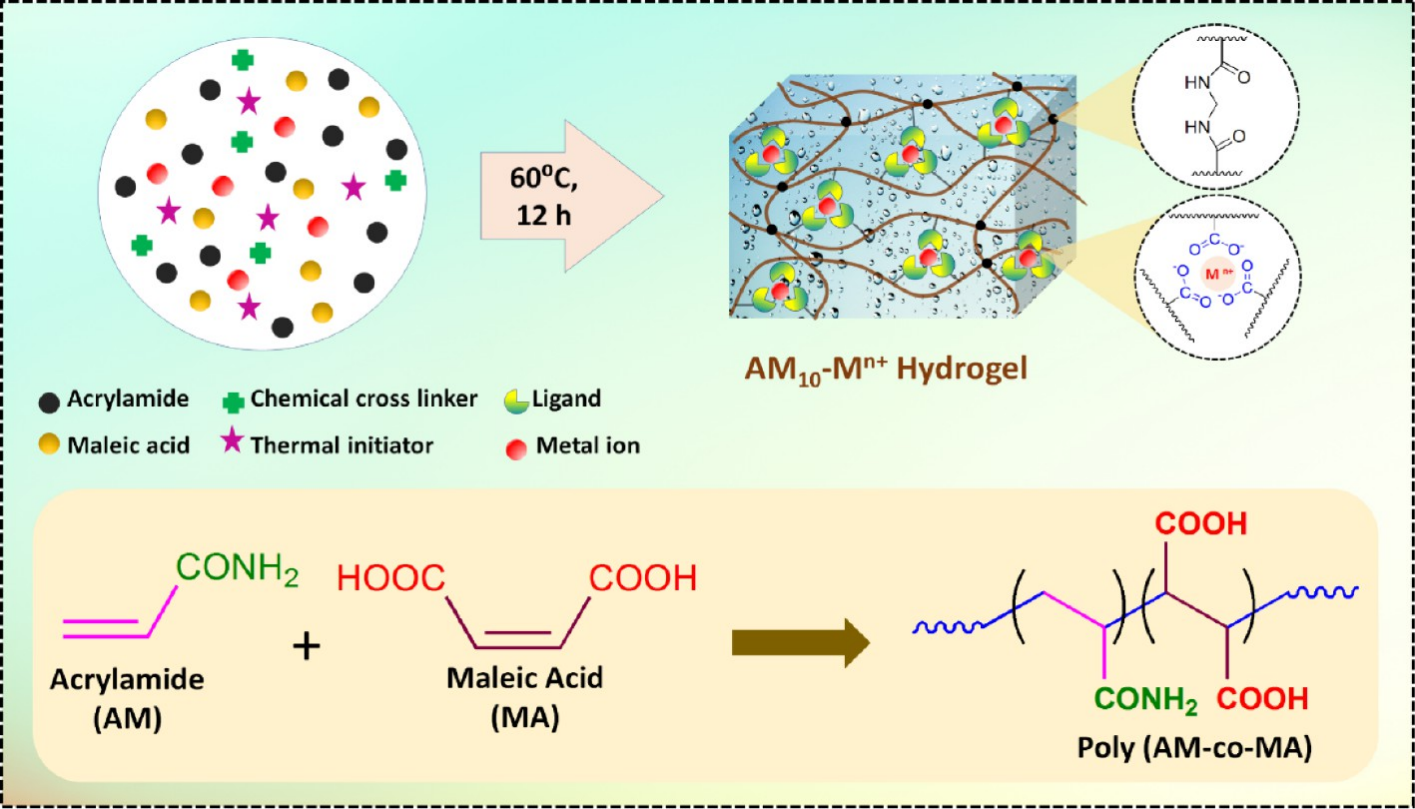
Synthesis of Metal Ion Crosslinking of Poly(AM-co-MA) Hydrogels and Depiction of Various Chemical Linking within the Hydrogel

In our current approach, we incorporated metal ions inside the hydrogel through in situ polymerization to prevent swelling. We used a set of different metal ions (Fe^3+^, Fe^2+^, Ca^2+^, Cu^2+^, Zn^2+^, Ni^2+^) and separately incorporated them with an aqueous solution mixture of two hydrophilic monomers, acrylamide (AM) and maleic acid (MA). Free radical thermal polymerization was carried out in the presence of a small amount of chemical cross-linker (MBAA) and ammonium persulfate (APS) initiator at 60 °C for 12 h (Scheme S1). Detailed compositions of the hydrogels are summarized in Table S1. Scheme 1 represents the basic design of the hydrogel. The dicarboxylic function of the maleic acid unit in this polymer chain coordinates with metal ions, and these physical cross-links act as sacrificial bonds to dissipate energy when the hydrogel is stretched. The chemical cross-links present at low density are expected to maintain structural integrity during deformation. Among the aforementioned metal ions, Ca^2+^, Ni^2+^, and Zn^2+^ formed in situ gel and the Ca^2+^ cross-linked hydrogel exhibited the highest mechanical strength. Under optimized conditions, the Ca^2+^ hydrogel showed a tensile strength of ∼250 kPa and a stretchability of ∼15–16 times its original length. Due to the presence of numerous H-bonding and Ca^2+^-carboxylate-based reversible cross-linking, the hydrogel showed a high fracture energy of ∼1500 J m^–2^, making it comparable to cartilage.^34^ Additionally, these reversible bonds also enhanced self-recovery, antifatigue, and self-healing properties. The inclusion of Ca^2+^ and other metal ions also made the hydrogel highly conductive, and this property was utilized to develop a strain sensor for detecting human motion. This hydrogel-based strain sensor was further used for detecting human motion and conveying the message through finger bending. The hydrogel was also used as a solid electrolyte in a flexible supercapacitor device, exhibiting not only high conductivity but also inhibiting liquid leakage and showing a specific capacitance of ∼260 F/g at 0.5 A/g current density. The Ca^2+^ cross-linked hydrogel’s high conductivity, along with its high water content entrapped in a highly porous structure, proved to be effective for absorption-dominant green EMI shielding properties. This hydrogel is intrinsically adhesive to a variety of substrates. This adhesiveness helps to strongly adhere with electrical connections and enhances the device performance. Simultaneously it could be used for covering the electronic devices for protecting them from electromagnetic interference, ensuring data safety and reducing the radiation hazards. The inclusion of Ca^2+^ not only enhanced these properties but also improved antifreezing and antidrying ability of these hydrogel materials, making them suitable for applications even at subzero temperature and ensuring long-term durability of the device. Overall, we have successfully explored diverse applications of this in situ metal complex hydrogel synthesized through a simple polymerization process.

## Results and Discussions

### Synthesis and Characterization of Hydrogels

**Figure 1:**
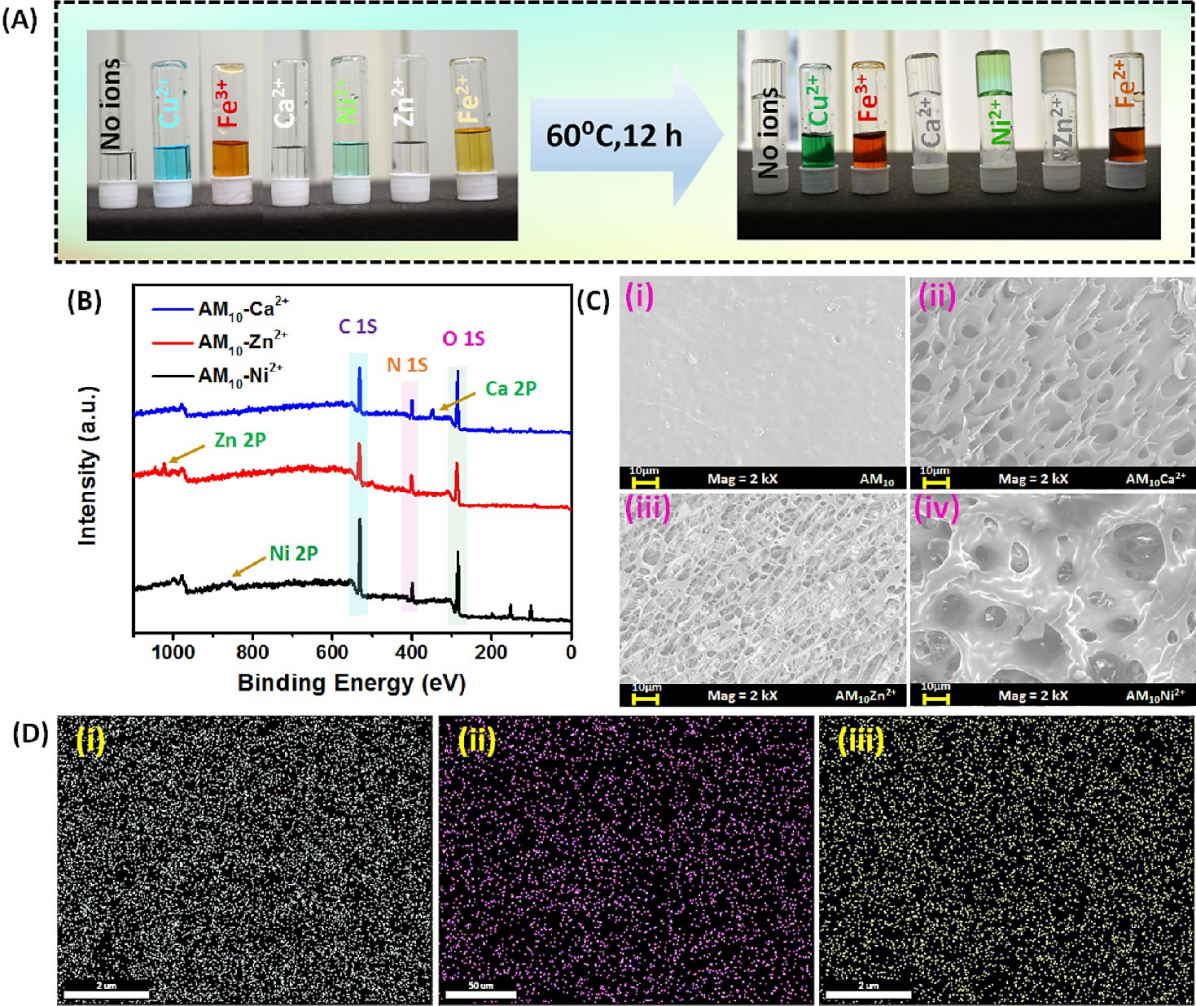
(A) In situ metal ion gel formation. (B) XPS spectroscopy of the AM_10_ hydrogel in the presence of Ca^2+^, Zn^2+^, and Ni^2+^ metal ions. (C) FESEM images of the AM_10_ hydrogel (i) in the absence of any metal ions and in the presence of (ii) Ca^2+^, (iii) Zn^2+^, (iv) Ni^2+^ metal ions. (D) Distribution of (i) Ca^2+^, (ii) Zn^2+^, and (iii) Ni^2+^ metal ions, revealed from EDS mapping in the AM_10_-M^n+^ hydrogel (where M^n+^ represents the metal ions, namely, Ca^2+^, Zn^2+^, and Ni^2+^).

A copolymer hydrogel was synthesized using acrylamide (AM) and maleic acid (MA) as a monomer, APS as a thermal initiator, and MBAA as a chemical cross-linker. In order to introduce metal–ligand interactions, different metal ions (Fe^3+^, Fe^2+^, Ca^2+^, Cu^2+^, Ni^2+^, Zn^2+^) have been incorporated in situ with the pregel solution mixture. The gel formation was carried out through thermal polymerization, which was conducted at 60 °C for 12 h. It was observed that except Fe^3+^, Fe^2+^, and Cu^2+^, all the other metal ions formed transparent hydrogel under in situ conditions (Figure 1A**)**. The metal ions formed a cross-linked structure with the dicarboxylic acid units of MA, which serve as a secondary physical cross-linker inside the hydrogel. Alongside, the acrylamide and MA units are also expected to have hydrogen-bonded cross-linking interactions. These metal-ion cross-linked hydrogels have been represented as AM_10_-M^n+^ (where maleic acid content 10 wt % represents the total monomer and M^n+^ denotes the metal ions). The XPS analysis demonstrated the presence of metal ions and other elemental compositions (Figure 1B). The characteristic peaks of C 1s, N 1s, and O 1s indicated the presence of C, N, and O atoms within the polymer in all cross-linked hydrogels. In addition, separate characteristic peaks for Ca 2p, Ni 2p, and Zn 2p were observed in AM_10_-Ca^2+^, AM_10_-Ni^2+^, and AM_10_-Zn^2+^ hydrogels, respectively, confirming the presence of metal ions in these hydrogels. FESEM analysis of the microstructure of the freeze-dried hydrogels revealed that the control hydrogel AM_10_ does not possess any porous microstructure. However, the metal ion cross-linked hydrogel displayed a porous microstructure, which can be attributed to the secondary cross-linking caused by the metal–ligand interaction (Figure 1C). The presence and distribution of metal ions within the hydrogel were further confirmed by EDS mapping (Figure 1D), which revealed a homogeneous distribution of Ca^2+^, Ni^2+^, and Zn^2+^ ions across the surface of the AM_10_-Ca^2+^, AM_10_-Ni^2+^, and AM_10_-Zn^2+^ hydrogels, respectively.

### Mechanical Properties

**Figure 2:**
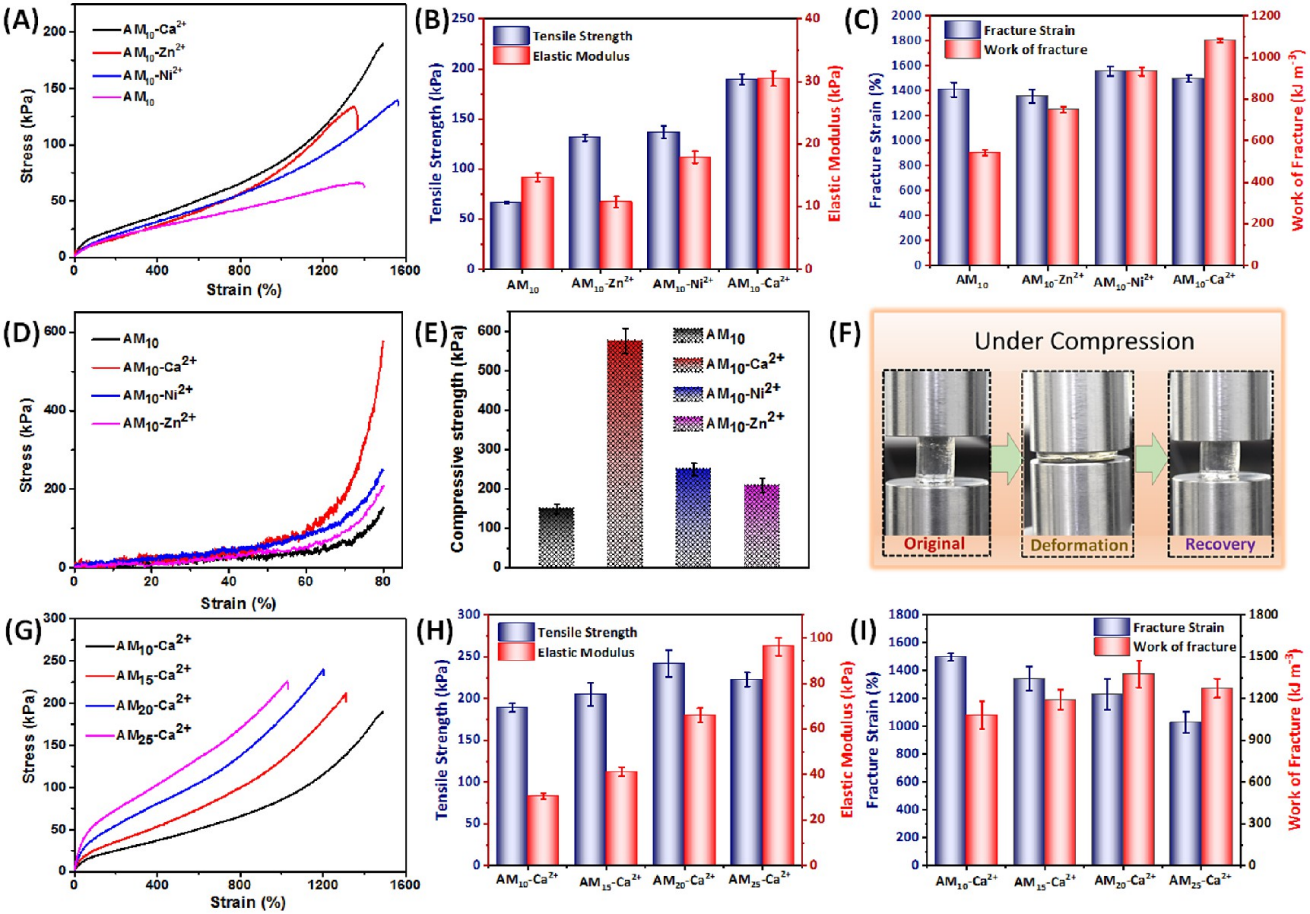
(A) Tensile stress strain diagram, (B) tensile strength and elastic modulus, (C) fracture strain and work of fracture, (D) compressive stress strain diagram, (E) compressive strength of AM_10_-Ca^2+^, AM_10_-Ni^2+^, AM_10_-Zn^2+^, and AM_10_ hydrogels. (F) Photograph of compressive deformation of AM_10_-Ca^2+^ hydrogels under compressive load and recovery of the AM_10_-Ca^2+^ gel upon removal of load. (G) Tensile stress strain diagram, (H) tensile strength and elastic modulus, (I) fracture strain and work of fracture of AM_*X*_-Ca^2+^ hydrogels (“*X*” represents different compositions of maleic acid. *X* = 10%,15%, 20%, and 25%, respectively).

To determine the effect of metal–ligand cross-linking on mechanical characteristics, the tensile stress–strain data for AM_10_ hydrogels were analyzed in the presence and absence of metal ions (Figure 2A). The molar ratio of maleic acid and the metal ions (Ca^2+^, Ni^2+^ and Zn^2+^) was maintained at 1:1 to ensure a stoichiometric charge balance. Among the various metal ions investigated, Ca^2+^ showed the greatest enhancement in the tensile strength and elastic modulus (Figure 2B). The inclusion of Ca^2+^ in the AM_10_-Ca^2+^ hydrogel resulted in a 3-fold increase in tensile strength (189.3 ± 5.3 kPa) compared to the control AM_10_ hydrogels (66.3 ± 1.1 kPa). Additionally, the tensile strength of the AM_10_-Ca^2+^ hydrogels was higher than that of the AM_10_-Ni^2+^ hydrogels (137 ± 5.9 kPa) and AM_10_-Zn^2+^ hydrogels (131.5 ± 3.3 kPa). The same trend was observed for the elastic modulus, with AM_10_-Ca^2+^ hydrogels (30.5 ± 1.2 kPa) exhibiting ∼2 times enhancement compared to AM_10_ hydrogels (14.7 ± 0.7 kPa), and the elastic modulus was also higher than that of the other metal ion-based hydrogels (AM_10_-Ni^2+^ hydrogel: 17.9 ± 0.9 kPa; AM_10_-Zn^2+^ hydrogel: 10.8 ± 0.9 kPa). These results suggest that Ca^2+^ has a stronger interaction with dicarboxylate ions compared to Ni^2+^ and Zn^2+^ ions, which is consistent with previous studies.^18^ Interestingly, there were no significant differences in breaking strain among the hydrogel samples, with each hydrogel able to stretch up to approximately 15–16 times its original length (Figure 2C). However, due to its high strength and stretchability, the AM_10_-Ca^2+^ hydrogel exhibited the highest work of fracture (1082 ± 11 kJ m^–3^), ∼2 times that of the control AM_10_ hydrogel (542 ± 14 kJ/m^3^), and also higher than the work of fracture for the AM_10_-Ni^2+^ hydrogel (932 ± 20 kJ m^–3^) and AM_10_-Zn^2+^ hydrogel (749 ± 15 kJ m^–3^).

Consistent with the tensile properties, the compressive properties of the hydrogels followed the same trend (Figure 2D). At 80% compressive strain, AM_10_ hydrogels exhibited a compressive strength of 149 ± 12 kPa. However, the inclusion of metal ions resulted in a significant increase in compressive strength, with AM_10_-Ca^2+^ hydrogels displaying a compressive strength of 576 ± 32 kPa, followed by AM_10_-Ni^2+^ hydrogels (250 ± 15 kPa) and AM_10_-Zn^2+^ hydrogels (209 ± 19 kPa) (Figure 2E). Notably, none of the hydrogels were broken at 80% compressive deformation, and all were able to recover their original shapes after the load was removed. This recovery of shape was attributed to the presence of reversible dynamic bonds, such as metal–ligand interactions and H-bonding. Under deformation, the dynamic metal–ligand cross-links dissociate but reassociate upon release of the load, allowing the hydrogels to restore their shape. Figure 2F shows the shape deformation of the AM_10_-Ca^2+^ hydrogel under compression and the shape recovery after releasing the load. Collectively, these results suggest that among the investigated metal complex-based hydrogels, the AM_10_-Ca^2+^ hydrogel exhibited the highest tensile and compressive properties. In order to optimize the concentration of the Ca^2+^ ions in the hydrogel, the mechanical properties of the AM_10_-Ca^2+^ hydrogels with different maleic acid/Ca^2+^ molar ratios were investigated. The results demonstrate that the hydrogel with a 1:1 molar ratio of maleic acid/Ca^2+^ showed the highest tensile strength (Figure S1 and Table S2), revealing this ratio to be optimal for subsequent detailed studies.

In addition to the various metal ions, the composition of the comonomer mixture utilized during polymerization is a critical factor in determining the mechanical characteristics of hydrogels. The influence of monomer concentration on hydrogel properties was investigated by varying the maleic acid concentration from 10 to 25 wt %, while maintaining a constant total monomer concentration (25 wt %). Hydrogels with differing maleic acid content were designated as AM_*X*_-Ca^2+^ (where “*X*″ denotes the maleic acid content, with *X* being 10, 15, 20, and 25 wt % of the total monomer). The maleic acid/Ca^2+^ molar ratio was maintained at 1:1 for all of the cases to maintain the stoichiometric charge balance of Ca^2+^ to carboxylate ions. These hydrogels were subjected to tensile experiments in order to evaluate their mechanical properties. Figure 2G illustrates the stress–strain plots for various hydrogels with different maleic acid contents. The results showed that with increasing maleic acid concentration, the mechanical strength gradually increased until reaching 20 wt %, after which it began to decrease. In contrast, the breaking strain continuously decreased with a higher maleic acid content. This can be attributed to the increase in carboxylic acid units, which results in more cross-linking points when combined with Ca^2+^, thereby enhancing the strength. However, beyond 20 wt % maleic acid content, the cross-linking density becomes too high, making the hydrogel brittle. The tensile strength reached 242 ± 16 kPa with a 20 wt % maleic acid content (Figure 2H). The elastic modulus continuously increased with increasing maleic acid content, reaching 96 ± 4 kPa with a variation of maleic acid content from 10 to 25 wt %. With increasing content of maleic acid, the breaking strain gradually decreased from 1498 ± 28% in the AM_10_-Ca^2+^ hydrogel to 1031 ± 76% in the AM_25_-Ca^2+^ hydrogel. The AM_20_-Ca^2+^ hydrogel showed the highest work of fracture (1377 ± 96 kJ m^–3^). Based on these results, the AM_20_-Ca^2+^ hydrogel was considered to be the optimized hydrogel due to its favorable mechanical properties.

### Fracture Energy

**Figure 3:**
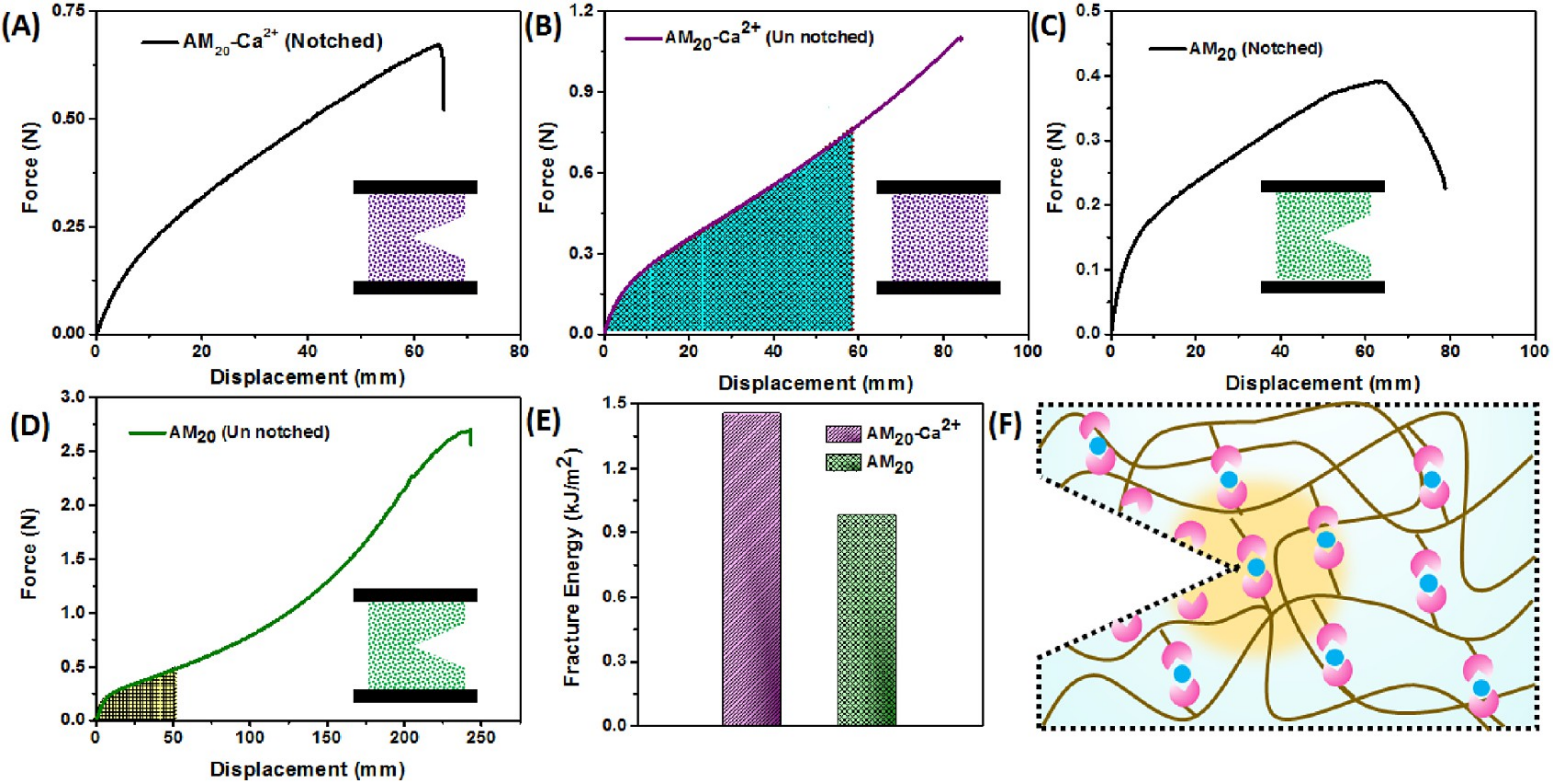
(A,B) Force–displacement curves of notched and unnotched AM_20_-Ca^2+^ hydrogels, respectively. (C,D) Force–displacement curves of notched and unnotched AM_20_ hydrogels, respectively. (E) Fracture energy of AM_20_-Ca^2+^ and AM_20_ hydrogels. (F) Schematic representation of crack propagation resistance in the presence of metal–ligand based cross-linking.

The AM_20_-Ca^2+^ hydrogel, with its abundant noncovalent metal–ligand interactions, has the potential to enhance the fracture energy of the hydrogel. The fracture energy of the AM_20_-Ca^2+^ hydrogel was determined by a method introduced by Rivlin and Thomas (pure-shear test),^35,36^ and compared to the fracture energy of the AM_20_ hydrogel without metal ion cross-linking. Accordingly, a notch (equivalent to 40% of the total width) was introduced to both the AM_20_-Ca^2+^ and AM_20_ hydrogel samples. Subsequently, these notched and unnotched hydrogel samples were subjected to a tensile experiment to measure their resistance to cracking. Figure 3A,B displays the force–displacement curves for the notched and unnotched AM_20_-Ca^2+^ hydrogel, while Figure 3C,D represents the corresponding plots for the AM_20_ hydrogel. The fracture energy of AM_20_-Ca^2+^ hydrogel (∼1.5 kJ m^–2^) was significantly higher than the fracture energy of the AM_20_ hydrogel (0.98 kJ m^–2^) (Figure 3E) as well as that of cartilage (1 kJ m^–2^).^34^ This high fracture energy can be attributed to the presence of additional noncovalent interactions, specifically dynamic metal–ligand cross-linking. In the case of AM_20_ hydrogels, the covalently bonded network bridges the crack and the rupture occurs due to localized damage, resulting in lower fracture energy, whereas in the case of AM_20_-Ca^2+^ hydrogels, the ionically cross-linked network unzips over a wide area, releasing the concentrated stress on the network surrounding the notch tip. This enables the energy stored in the chains to be transferred to a larger zone (represented by yellow in Figure 3F), consequently leading to a higher fracture energy. Crack blunting at a high stretch was also demonstrated by the AM_20_-Ca^2+^ hydrogel (Figure S2).

### Energy Dissipations under Cyclic Loading and Unloading Test

**Figure 4:**
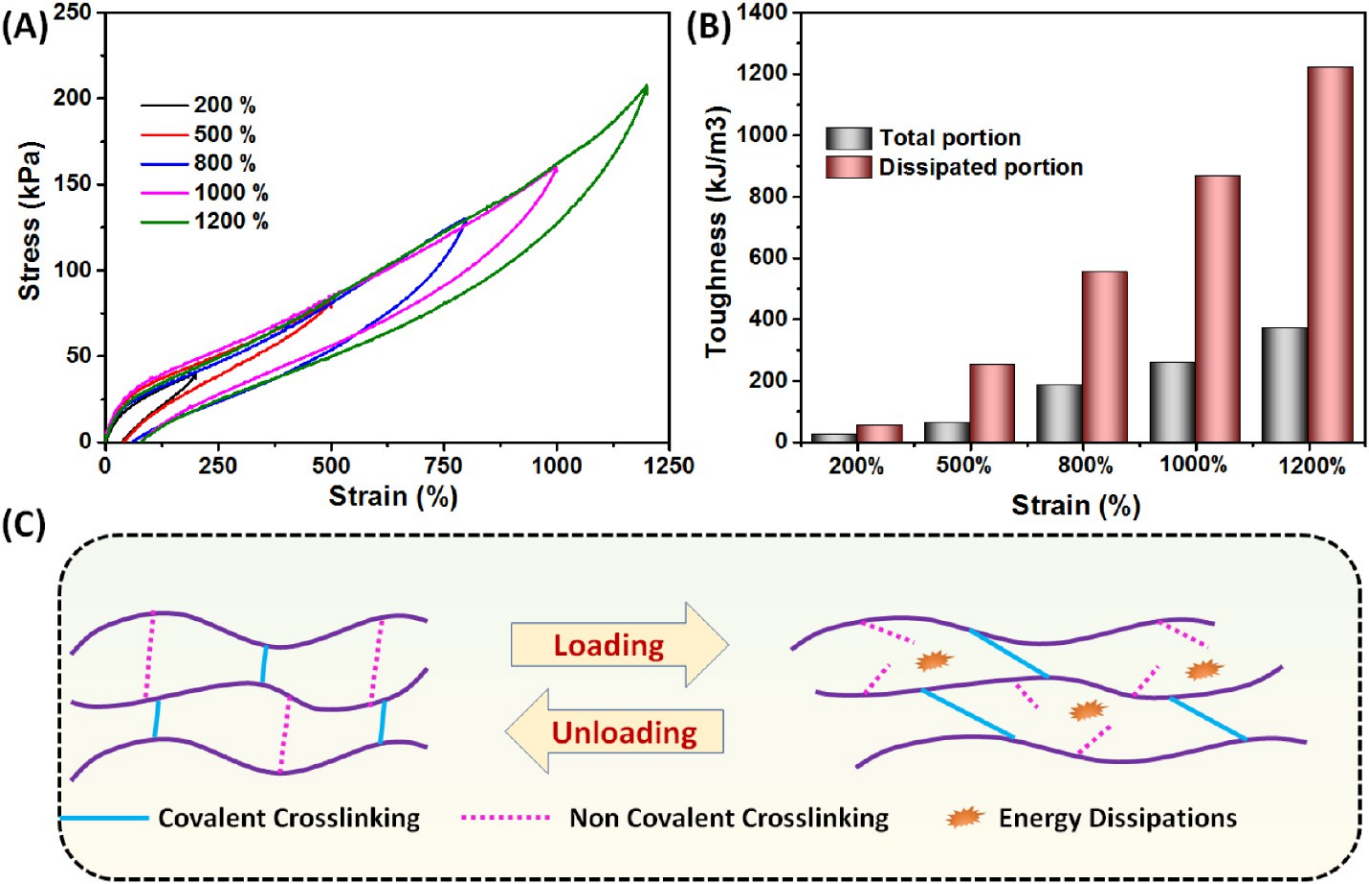
(A) Cyclic tensile loading and unloading experiments of the AM_20_-Ca^2+^ hydrogel. (B) Dissipated energy under the hysteresis loop and total energy dissipation during cyclic loading and unloading experiments at different strain %. (C) Energy dissipation mechanism of the AM_20_-Ca^2+^ hydrogel through the breaking of dynamic noncovalent cross-links.

The AM_10_-Ca^2+^ hydrogel was subjected to multiple cycles of tensile loading and unloading up to various tensile strains, as illustrated in Figure 4A. During these cycles, the hydrogel showed a significant hysteresis region indicative of the amount of energy dissipated during cyclic deformation. The amount of energy dissipated for the tensile loading–unloading cycle to 200% strain was ∼22 kJ m^–3^, which accounted for ∼42% of the total work (as determined by the area under the loading curve, ∼52 kJ m^–3^) (Figure 4B). This substantial dissipation of energy can be attributed to the rupture of sacrificial bonds present in the hydrogel network. Figure 4C schematically represents the energy dissipation mechanism of the AM_10_-Ca^2+^ hydrogel. The AM_10_-Ca^2+^ hydrogel contains Ca^2+^-carboxylate coordination bonds and hydrogen bonds, which serve as energy dissipating motifs. As the strain percentage increased, there was a gradual increase in the amount of energy dissipated. At a strain of 1200%, the dissipated energy was ∼0.4 MJ m^–3^, which was 30% of the total work (1.2 MJ m^–3^). This was ∼17 times higher compared to the dissipation of energy at a strain of 200%. The trend suggests that higher levels of strain resulted in a larger percentage of sacrificial bond breakage, leading to a significant increase in the energy dissipation. The impressive mechanical robustness of the AM_10_-Ca^2+^ hydrogel can be attributed to its high energy dissipations and toughness. This is demonstrated in Figure S3, where the hydrogel strip was subjected to stretching (Figure S3A), bending (Figure S3B), compressing (Figure S3C), and twisting (Figure S3D). The hydrogel was also puncture resistant (Figure S3E).

### Self-Recovery and Antifatigue Characteristics

**Figure 5:**
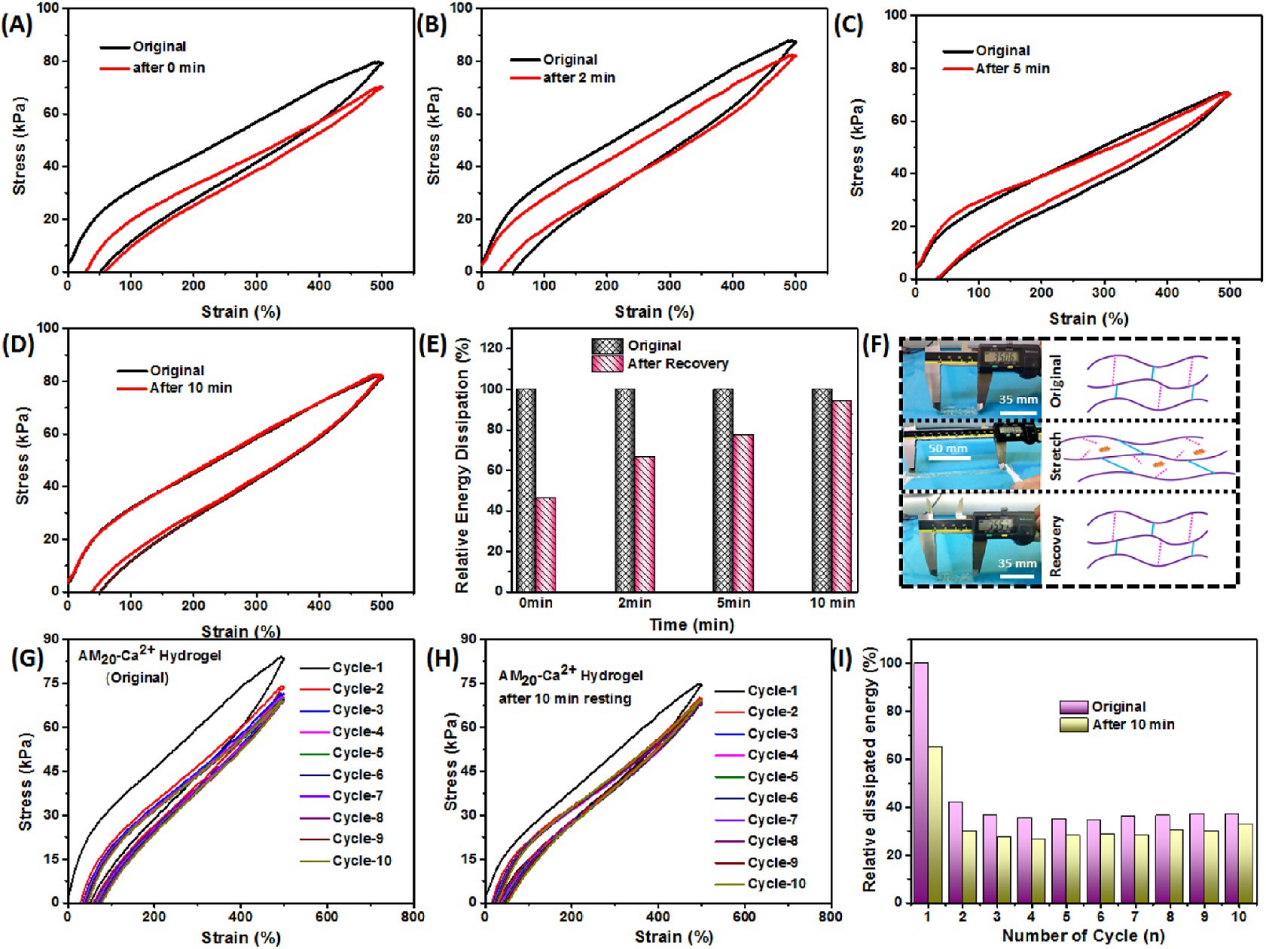
Cyclic tensile loading–unloading experiments of the AM_20_-Ca^2+^ hydrogel: (A) Self-recovery at 0 min, (B) self-recovery at 2 min, (C) self-recovery at 5 min, and (D) self-recovery at 10 min. (E) Relative energy dissipation when the second loading cycle was started at different time intervals. (F) Photograph of the AM_20_-Ca^2+^ hydrogel that underwent cyclic deformation and recovers to its original state. (G) Ten successive loading and unloading cycles during antifatigue test. (H) After resting for 10 min, further ten successive loading and unloading cycles during antifatigue test. (I) Relative recovery of dissipated energy of ten successive loading and unloading cycles of original and self-recovered samples after resting for 10 min.

Although many conventional hydrogels dissipate significant amount of energy during deformation, they cannot recover that energy quickly upon removal of the load, which is an inherent disadvantage when utilized in repeated load-bearing applications.^24,37,38^ The AM_20_-Ca^2+^ hydrogelis expected to show good self-recovery due to the abundance of many dynamic bonds, such as metal–ligand interactions and H-bonding. The self-recovery properties of the AM_20_-Ca^2+^ hydrogel were assessed by performing cyclic loading and unloading experiments up to 500% strain. After the first tensile loading–unloading cycle, the hydrogel exhibited ∼51% residual strain, which decreased to around 29% when the second loading–unloading cycle began immediately after the first (Figure 5A). The hysteresis areas of the two cycles differed significantly, indicating that the hydrogel was unable to fully recover its dissipated energy immediately after the first loading–unloading cycle. However, the amount of recovery was improved when the hydrogel was allowed to rest for a period of time. Resting the hydrogel for 2 min after the first loading–unloading event resulted in no residual strain (Figure 5B). Additionally, as the resting time increased from 2 to 5 to 10 min, the recovery of the hydrogel increased (Figure 5B–D) due to the additional time for the broken sacrificial bonds to reconnect. The extent of recovery was quantitatively measured by comparing the hysteresis area under the first and second cyclic loading data for each resting time (Figure 5E). It was found that only 46% of the dissipated energy was recovered when the second loading was applied immediately after the first loading–unloading event. However, the hydrogel was able to recover 94% of its dissipated energy after 10 min of rest (Figure 5E). This increased recovery with longer resting times can be attributed to the formation of a higher number of sacrificial bonds (Ca^2+^-carboxylate complex-based metal–ligand cross-links and H-bonds). Figure 5F visually and schematically illustrates the self-recovery properties of the AM_20_-Ca^2+^ hydrogel. When a strip of the hydrogel is stretched, the sacrificial bonds break and dissipate energy. Upon relaxation, the hydrogel promptly returns to its original length. This phenomenon is attributed to the dynamic nature of the sacrificial bonds, which rejoin after the hydrogel relaxes and recovers its original structure. To further investigate its potential as a fatigue-resistant material, the hydrogel was subjected to ten consecutive loading–unloading cycles (Figure 5G**)**. A significant decrease in the dissipated energy (hysteresis area) after the first cycle indicated that the hydrogel was unable to fully recover its dissipated energy. This can be attributed to the rupture of Ca^2+^-carboxylate bonds during loading and the lack of sufficient time for these bonds to reassociate in between cycles. The hydrogel was allowed to rest at ambient conditions for 10 min before undergoing another ten cycles (Figure 5H). This led to a recovery of ∼65% of the dissipated energy from the initial cycle (Figure 5I), demonstrating the robust antifatigue characteristics of the AM_20_-Ca^2+^ hydrogel. The significant recovery of energy within 10 min after ten consecutive loading–unloading cycles showcases the hydrogel’s sustainability under repeated loading conditions. Overall, these findings demonstrate the robust antifatigue properties of the AM_20_-Ca^2+^ hydrogel, with its ability to sustain under consecutive loading–unloading cycles and recover a significant amount of energy and tensile strength within a short resting time. This highlights its potential for load-bearing applications and emphasizes the importance of incorporating antifatigue properties in the development of mechanically resilient hydrogels.

### Adhesive Properties

**Figure 6:**
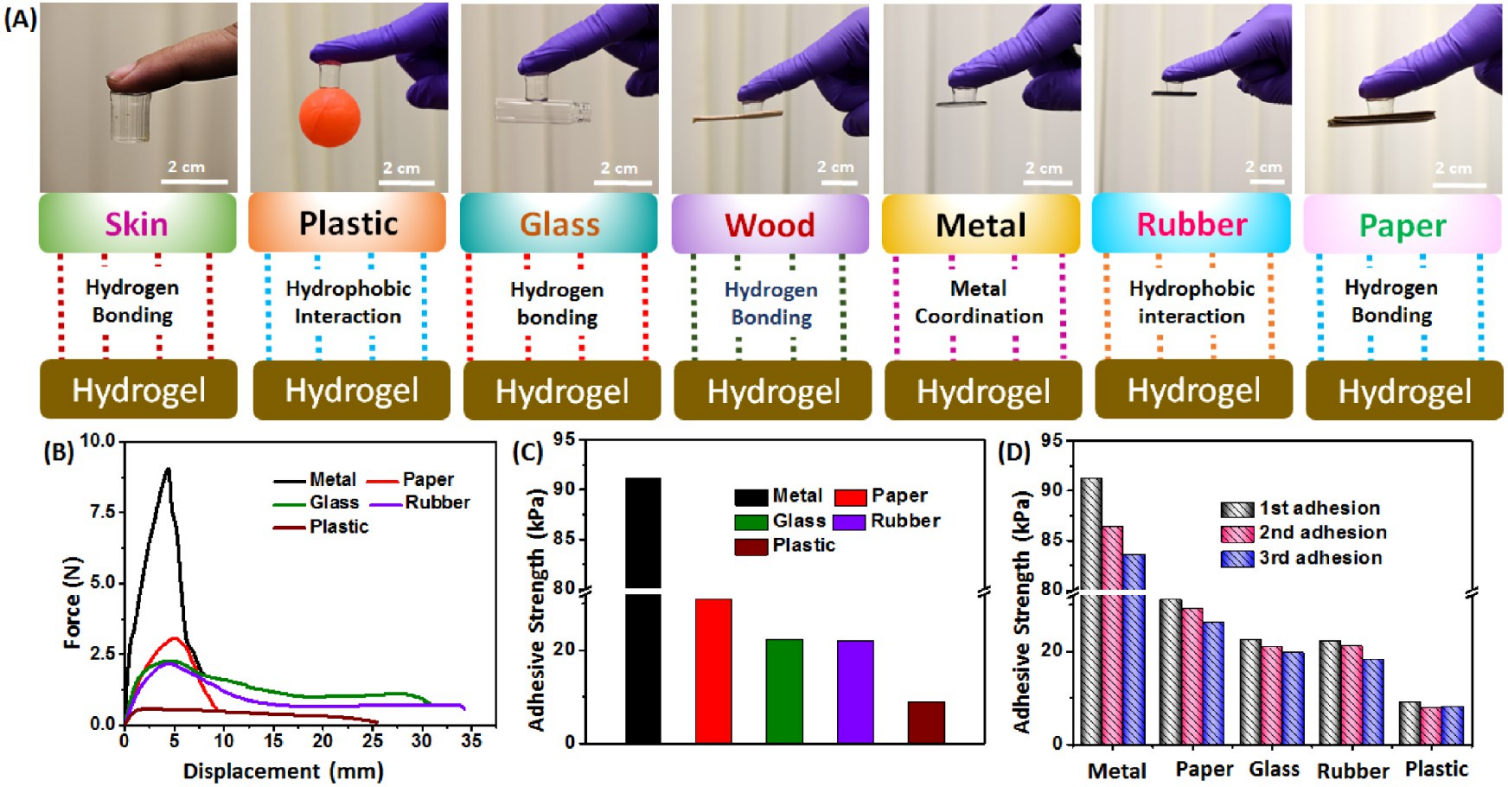
(A) Adhesion of the AM_20_-Ca^2+^ hydrogel with different substrates like skin, plastic, glass, wood, metal, rubber and paper. (B) Force vs displacement graphs for lap shear tests. (C) Adhesive strengths on different surfaces. (D) Repeated adhesion strength of the AM_20_-Ca^2+^ hydrogel with different substrates like metal, paper, glass, rubber, and plastic.

In order to effectively utilize hydrogels for a variety of purposes, it is important for the material to possess strong adhesive properties on different types of surfaces. An investigation of the adhesive characteristics of the hydrogel on various substrates was undertaken. Due to the presence of functional groups such as carboxylic acid and amide, it was expected that the AM_20_-Ca^2+^ hydrogel would exhibit strong adhesive properties. Indeed, the AM_20_-Ca^2+^ hydrogel can adhere to various surfaces including human skin, plastic, glass, wood, metal, rubber, and paper (Figure 6A).^39,40^ The adhesion mechanism of hydrogels with the human skin is attributed to the hydrogen bonding and electrostatic interaction of a protein molecule in tissue with the carboxyl groups of the hydrogel. These hydrogels adhere to the polypropylene plastic substrate and nitrile rubber substrate through hydrophobic interactions.^41−43^ Besides, strong adhesion of hydrogels with glass, wood, and paper occurs through the H-bonding interactions. The adhesive strength of AM_20_-Ca^2+^ was quantitatively assessed through a lap shear test on metal, glass, rubber, and plastic substrates. The resulting force–displacement curve was used to determine the adhesion strength. The adhesion strengths for metal (aluminum sheet), paper, glass, rubber, and plastic were found to be approximately 91, 31, 22, 22, and 9 kPa respectively (as shown in Figure 6B,C). The adhesion strength of AM_20_-Ca^2+^ with the metal substrate (aluminum sheet) was likely to be the strongest due to the strong coordination interactions between the pendant ligand groups (carboxylic acid) and the metal surface. After repeated adhesions to the respective surfaces for three cycles, the adhesion strength decreased but still remained significant compared to the original adhesion strength (Figure 6D), demonstrating the multitime adhesion capabilities of the hydrogel and indicating that adhesion occurred through the attachment and detachment of dynamic reversible bonds. The high adhesive strength and repeated adhesion also indicate that the hydrogel can easily adhere to various types (hydrophilic or hydrophobic) of surfaces.

### Antifreezing and Antidehydration Properties

**Figure 7:**
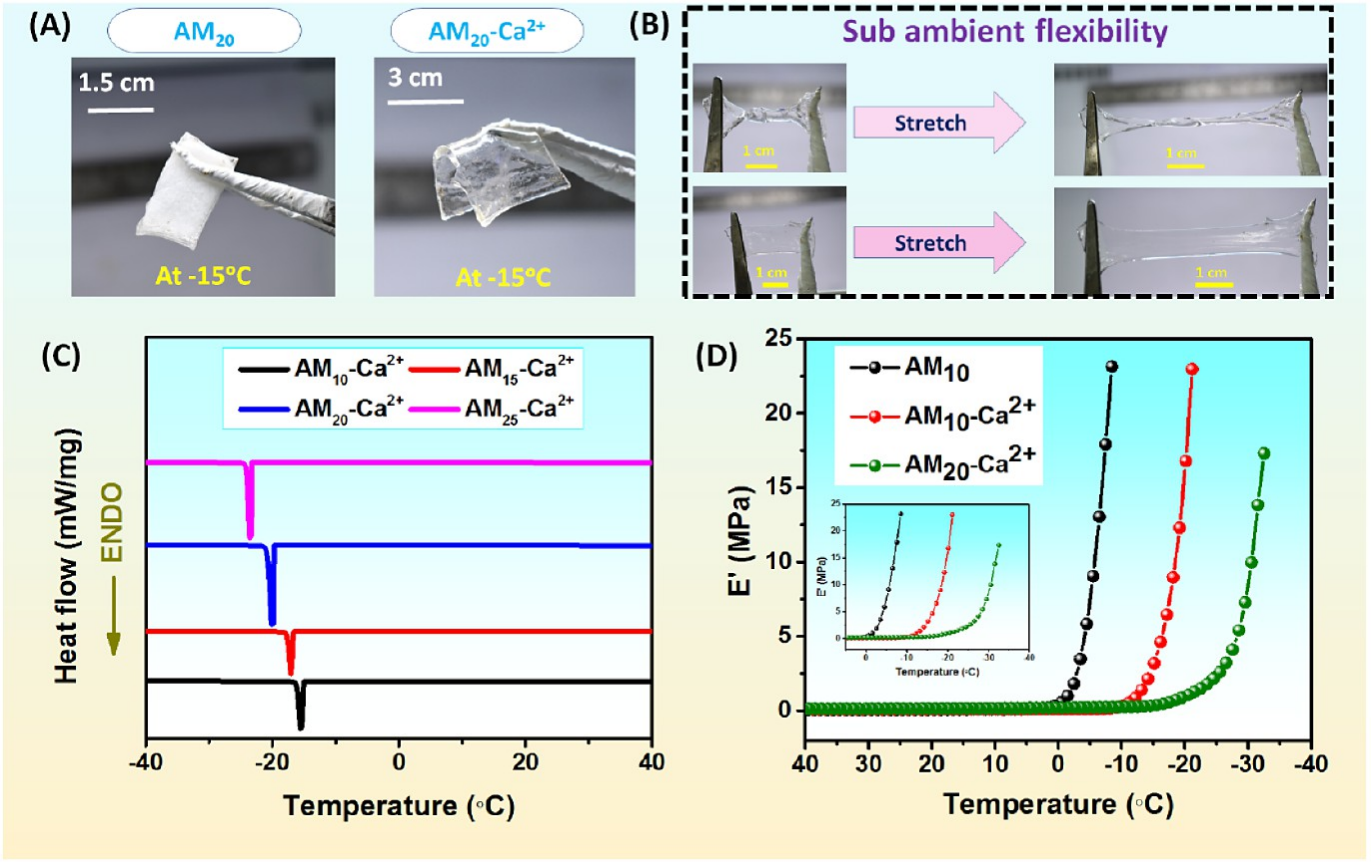
(A) Photographs of AM_20_ and AM_20_-Ca^2+^ hydrogels after keeping at −15 °C for 24 h. After 24 h, AM_20_ lost its flexibility, whereas the AM_20_-Ca^2+^ hydrogel still maintained its flexibility due to its antifreezing properties. (B) Photograph of the AM_20_-Ca^2+^ hydrogel after keeping at −15 °C for 24 h. It can be twisted and can be stretched. Also, the hydrogel was stretched 5 times of its original length without breaking, representing the flexible nature at subzero temperatures. (C) DSC experiments of the AM_*x*_-Ca^2+^ hydrogel (where *X* = 10%,15%, 20%, and 25%). (D) DMA experiments of AM_10_, AM_10_-Ca^2+^, and AM_20_-Ca^2+^ hydrogels.

A common challenge faced by conventional hydrogels is their lack of antifreezing and antidehydration properties. Most conventional hydrogels tend to freeze when exposed to subzero temperatures, thus losing their flexibility. However, the presence of Ca^2+^ ions can weaken the hydrogen bonds between water molecules, disrupt the formation of water molecule aggregates, and effectively impede the formation of ice crystals in the hydrogel,^44^ which prevents freezing of the hydrogel at subzero temperatures. Biological organisms residing in extremely cold environments, for instance, the Antarctic bacterium *Marinomonas primoryensis*, has been documented to depend on Ca^2+–^dependent antifreeze protein (AFP) for survival.^45^ Indeed, the AM_20_-Ca^2+^ hydrogel is able to maintain its flexibility even at temperatures as low as −15 °C. After 24 h at −15 °C, the AM_20_ hydrogel film is frozen and rigid, while the AM_20_-Ca^2+^ hydrogel remains flexible (Figure 7A). Additionally, Figure 7B highlights the remarkable subzero temperature flexibility of the AM_20_-Ca^2+^ hydrogel, showcasing its ability to withstand twisting to a significant degree at low temperatures. In contrast, the AM_20_ does not exhibit any subambient temperature flexibility, emphasizing the importance of the presence of Ca^2+^ ions in its antifreezing properties. To precisely study the change of freezing temperature of the hydrogel, differential scanning calorimetry (DSC) experiments were conducted over a temperature range of −40 to 40 °C. As the maleic acid (MA) content increased, the endothermic peak associated with the melting of bound ice crystals gradually shifted to lower temperatures (Figure 7C). For the AM_10_-Ca^2+^ hydrogel, the endothermic peak appeared at approximately −15 °C, but as the MA concentration increased to 15 wt % (AM_15_-Ca^2+^ hydrogel), 20 wt % (AM_20_-Ca^2+^ hydrogel), and 25 wt % (AM_25_-Ca^2+^ hydrogel), the peak shifted to approximately −17 °C, −20 °C, and −24 °C, respectively. The addition of CaCl_2_ into the hydrogel system in a stoichiometric ratio with MA resulted in an increase in the amount of CaCl_2_, causing the freezing point to shift toward a lower temperature as the MA content increased. The difference in freezing points was visually observed when the hydrogels with various MA contents were placed in a deep freezer (held at approximately −20 °C). The hydrogel without any Ca^2+^ froze within 3 h, while the AM_10_-Ca^2+^ hydrogel took around 8 h to freeze, followed by the AM_15_-Ca^2+^ hydrogel (∼12 h), and finally the AM_20_-Ca^2+^ hydrogel (∼6 days) (Figure S4). It was also observed that the AM_25_-Ca^2+^ hydrogel did not freeze, even after 6 days. So, it is clear that the change in MA concentration directly impacts the freezing time due to the variation in Ca^2+^ ion levels caused by the alteration in the MA content. The antifreezing properties were further confirmed using dynamic mechanical analysis (DMA) experiments carried out from 50 °C to −30 °C (Figure 7D). On cooling the hydrogels, it was observed that the compressive storage modulus (*E*′) of the AM_10_ hydrogel shows a sharp increase at ∼0 °C, indicating freezing of the hydrogel at this temperature. For the AM_10_-Ca^2+^ hydrogel, *E*′ begins to rise sharply at approximately −8 °C, indicating that the AM_10_-Ca^2+^ hydrogel has antifreezing properties attributed to the presence of Ca^2+^ ions. For the AM_20_-Ca^2+^ hydrogel, freezing occurs at a lower temperature (−18 °C) compared to AM_10_-Ca^2+^, attributed to the higher concentration of Ca^2+^ ions. In absence of Ca^2+^, water molecule forms H-bonding and agglomerates, whereas in the presence of Ca^2+^ it interacts with the water molecules and restricts the water molecule to form H-bonding, which lowers the freezing point of the trapped water inside the hydrogel. Hence, the hydrogel can retain its flexible and stretchable nature even at subzero temperatures.

One major challenge facing traditional hydrogels is their tendency to dehydrate, resulting in storage difficulties and deterioration of their functional properties over time. However, the incorporation of CaCl_2_ salt and utilization of Ca^2+^ ions in the AM_20_-Ca^2+^ hydrogel greatly enhanced its ability to resist dehydration.^44,46^ The water loss of the AM_20_-Ca^2+^ hydrogel when exposed to open air was compared to that of the AM_20_ hydrogel (Figure S5A). It was observed that the rate and amount of water loss of the AM_20_-Ca^2+^ hydrogel was significantly lower than the AM_20_ hydrogel in open air. After 2 days at 30 °C, the AM_20_-Ca^2+^ hydrogel lost ∼47% of its total water content, while the AM_20_ hydrogel lost around ∼65%. Additionally, when placed in a closed environment at a constant humidity (∼75%) and 30 °C, after 30 days the AM_20_-Ca^2+^ hydrogel only lost ∼16% of its water content, while the AM_20_ hydrogel lost around ∼55% (Figure S5B). This remarkable retention can be attributed to the unique combination of CaCl_2_ and hydrogel, which creates a lower vapor pressure inside the hydrogel and allows the CaCl_2_ to absorb water, making the hydrogel highly resistant to dehydration. Thus, the exceptional freezing and dehydration resistance of the AM_20_-Ca^2+^ hydrogel make it ideal for use under extreme conditions.

### Self-Healing Properties

**Figure 8:**
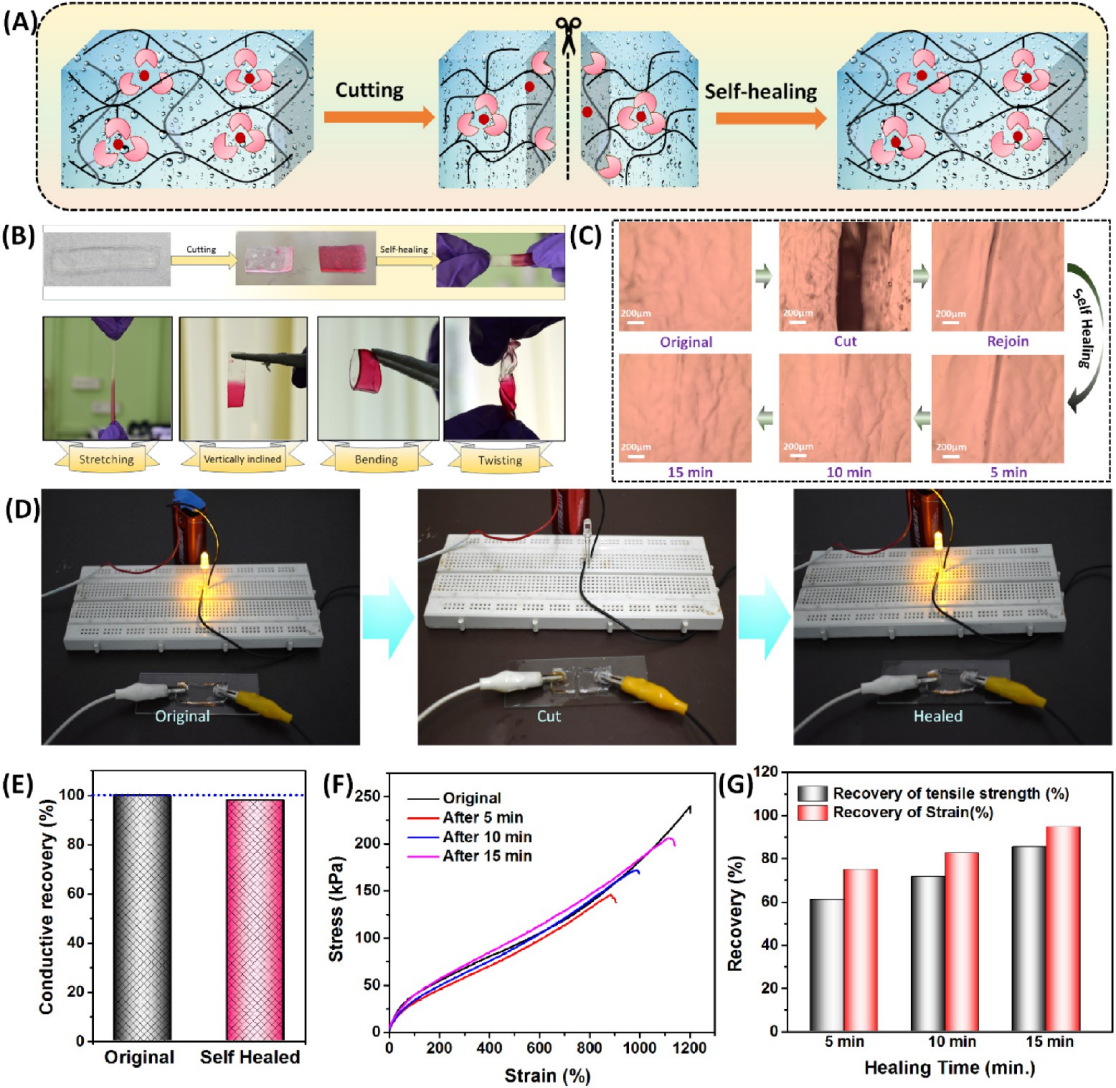
(A) Schematic representation of the self-healing mechanism in the hydrogel. (B) Self-healing process of the AM_20_-Ca^2+^ hydrogel. A rectangular piece of hydrogel was cut into two halves, one part stained with dye, then rejoined and kept for some time for self-healing. The self-healed gel could be stretched, vertically aligned, bent, and twisted without delamination at the healed surface. (C) Optical microscopy images of the hydrogel taken at different time points of self-healing. (D) The DC conductivity of the hydrogel can be restored, and it can light up an LED bulb after self-healing. (E) Recovery of conductivity after self-healing. (F) Stress–strain diagram of the AM_20_-Ca^2+^ hydrogel at different healing times. (G) % Recovery of tensile strength and breaking strain at different intervals of time.

The hydrogel, composed of many reversible cross-linked bonds such as metal–ligand and hydrogen bonded cross-links, is anticipated to possess self-healing capabilities. Figure 8A schematically represents the self-healing mechanism. The broken metal–ligand cross-links should be able to reassociate across the damaged surfaces of two pieces of hydrogel, leading to self-healing. To demonstrate its self-healing properties, we cut the hydrogel into two pieces. The pieces were then pressed together and left at room temperature for different time intervals (5, 10, and 15 min) (Figure 8B). To differentiate the two halves, one of the cut portions was stained, and it was observed that the hydrogel was able to self-heal immediately after rejoining. The healed sample could be stretched, bent, and twisted without delamination at the cutting site (Figure 8B). This represents the hydrogel’s excellent self-healing qualities. The self-healing process could be visually observed by investigation under an optical microscope. When the two pieces of hydrogel (cut from one hydrogel slab) were pressed together, the damage completely disappeared within 15 min and the hydrogel healed across the damaged surfaces (Figure 8C). This can be attributed to the increased number of broken bonds rejoining over a longer duration, resulting in the disappearance of the cut marks and restoration of mechanical properties. When the healed hydrogel was connected to a 9 V battery through a circuit, it was able to successfully illuminate an LED bulb, demonstrating the restoration of its conductivity (Figure 8D). The self-healed hydrogel was able to recover ∼98% of its DC conductivity within 1 min of rejoining (Figure 8E). The self-healing efficiency of the AM_20_-Ca^2+^hydrogel was quantitatively evaluated by measuring its mechanical properties after various time intervals (Figure 8F). The results showed that within 5 min of rejoining the hydrogel pieces, the material recovered ∼61% of tensile strength and ∼75% of breaking strain. After 15 min of waiting, the recovery of the tensile strength increased to 86% and the recovery of the breaking strain was enhanced to 95% (Figure 8G). Taken together, these results demonstrate the efficient and fast self-healing characteristics of the AM_20_-Ca^2+^hydrogel.

### Conductive Properties

**Figure 9:**
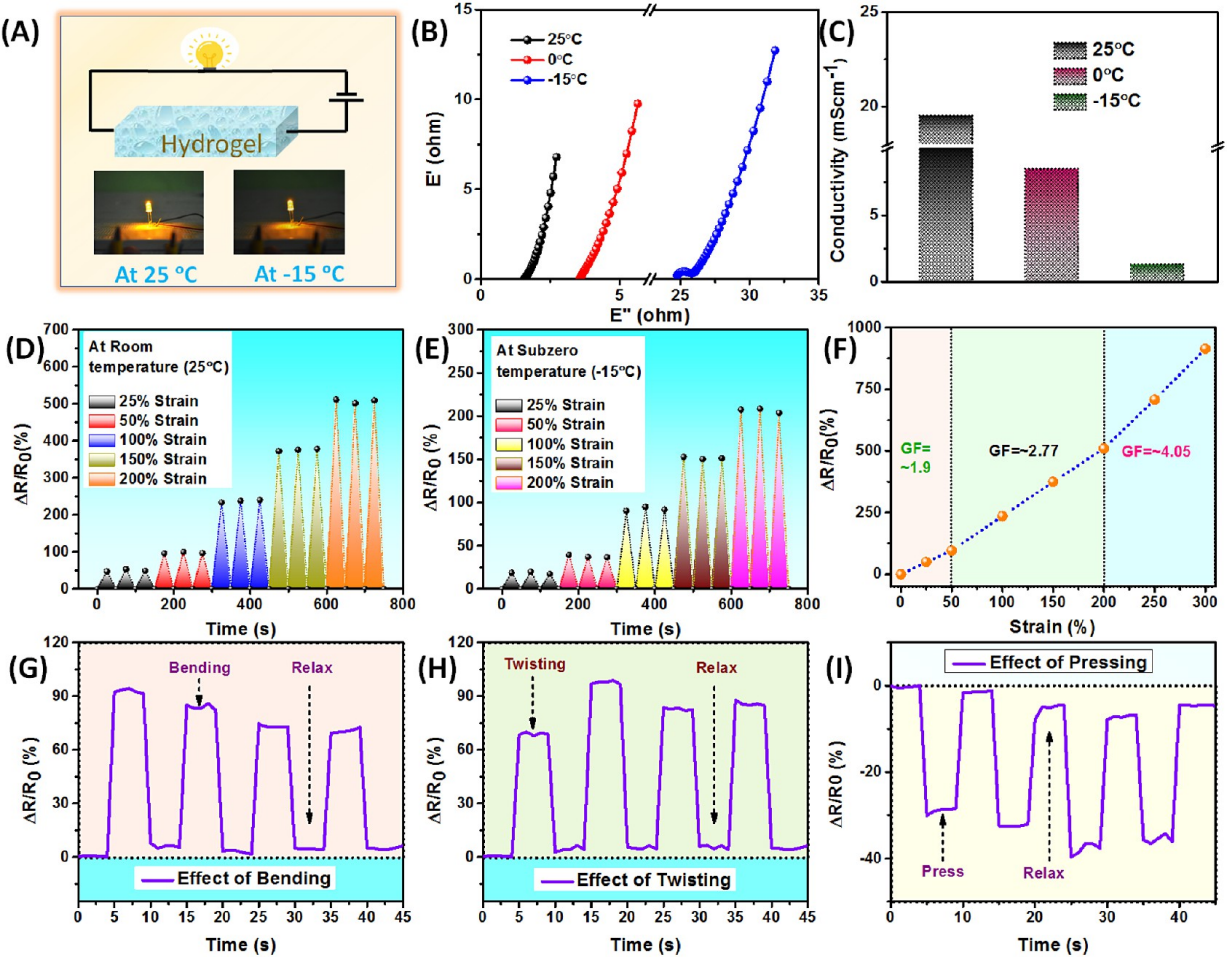
(A) Demonstration of conductive nature of the AM_20_-Ca^2+^ hydrogel at room temperature (25 °C) and subambient temperature (−15 °C). Connecting with a battery (using the mentioned circuit), the hydrogel was able to light up an LED both in ambient and subambient environments. (B) The Nyquist plots of the AM_20_-Ca^2+^ hydrogel at different temperatures (at 25, 0, and −15 °C). (C) Ionic conductivity of the AM_20_-Ca^2+^ hydrogel at different temperatures (at 25, 0, and −15 °C). Relative resistance change of the AM_20_-Ca^2+^ hydrogel as a function of strain (D) at ambient temperature (25 °C) and (E) at −15 °C. (F) Relative resistance change and gauge factor of the AM_20_-Ca^2+^ hydrogel during stretching the hydrogel at ambient temperatures. (G) Relative resistance change of the AM_20_-Ca^2+^ hydrogel during (G) bending, (H), twisting, and (I) pressing.

The presence of metal ions is expected to make this gel ionically conducting. In order to investigate this conductive property, a rectangular AM_20_-Ca^2+^ hydrogel sample (10 mm × 5 mm × 2 mm) was connected to a LED bulb using a 9 V battery, resulting in the emission of bright light (Figure 9A). The incorporation of Ca^2+^ ions not only enhanced the mechanical strength and conductivity of the gel but also provided antifreezing properties. As a result, this hydrogel remained flexible and did not freeze below 0 °C. However, at lower temperatures, the intensity of the LED light dimmed due to a decrease in conductivity (Figure 9A). To measure the conductivity of the AM_20_-Ca^2+^ hydrogel, electrochemical impedance spectroscopy (EIS) experiments were conducted at room temperature and also after keeping the hydrogel for 12 h at 0 °C and −1 5 °C, and the results showed a decrease in ionic conductivity due to restricted ionic movement at lower temperatures (Figure 9B). The AM_20_-Ca^2+^ hydrogel exhibited ionic conductivity of ∼19.5 mS cm^–1^ at room temperature (25 °C). The ionic conductivity decreased to ∼8.5 mS cm^–1^ at 0 °C and to ∼1.2 mS cm^–1^ at −15 °C (Figure 9C). The conductivity of the hydrogel was strain dependent. When the hydrogel was stretched, the LED light intensity dimmed (Video S1). This quality makes the hydrogel a promising candidate for strain-sensing applications. To construct a strain sensor, a rectangular strip of the AM_20_-Ca^2+^ hydrogel was placed between two stretchable layers made of VHB tape with metal wires connected to the hydrogel. The hydrogel’s strain-sensing ability was examined by subjecting it to consecutive cycles of tensile loading and unloading at various strains, during which the corresponding change in resistance was measured. It was noted that when the hydrogel was subjected to a specific strain percentage under cyclic loading and unloading, the relative resistance change remained consistent across multiple cycles. This demonstrated the stability of the resistive sensor, which is possibly due to the efficient self-recovery capability of the hydrogel. At both ambient and subzero temperatures (−15 °C), there was a quantitative change in relative resistance with increasing strain percentages (Figure 9D,E). These results indicate that this hydrogel material can be used as a flexible strain sensor both at ambient as well as subzero temperatures. However, compared to the ambient temperature, relative resistance change at subambient temperatures is lower due to the slower ionic transportation.^47,48^

The remarkable flexibility of the hydrogel enables it to be stretched to varying levels of strain, which was evident in the increase of the relative resistance change as the strain percentage increased. To measure the efficiency of the sensor, the gauge factor (defined as the change in relative resistance per unit strain) was calculated. In the low strain region (0–50%), the gauge factor was 1.9, but as strain percentage reached 200% and 300%, the gauge factor rose to 2.77 and 4.05, respectively (Figure 9F). These values are higher than the gauge factor of some other conventional reported hydrogels used for strain-sensing applications such as, double network hydrogels (GF is 0.2–0.3 at 100%),^49^ Fe^3+^ cross-linked (PVA–PAA-CNT) hydrogel,^50^ SWCNT/hydrogel (GF 0.25 at 100% strain),^51^ poly(AM-*co*-MA)/Fe^3+^ (GF 2.2 at 100% strain),^24^ PAA/BA/Fe^3+^/NaCl hydrogel (GF 2.48 at 400%).^52^

The strain-sensing ability of this hydrogel was further analyzed by subjecting it to bending, twisting, and pressing (Figure 9G–I). Results showed an increase in relative resistance change during bending and twisting, with a return to the original state after relaxing. This enhancement in the relative resistance change is due to the stretching of the hydrogel during bending and twisting. However, when subjected to pressing, the relative resistance change was observed to decrease as the distance between two connections reduced, allowing ions to move at a faster rate.

### Human Movement Detection

**Figure 10:**
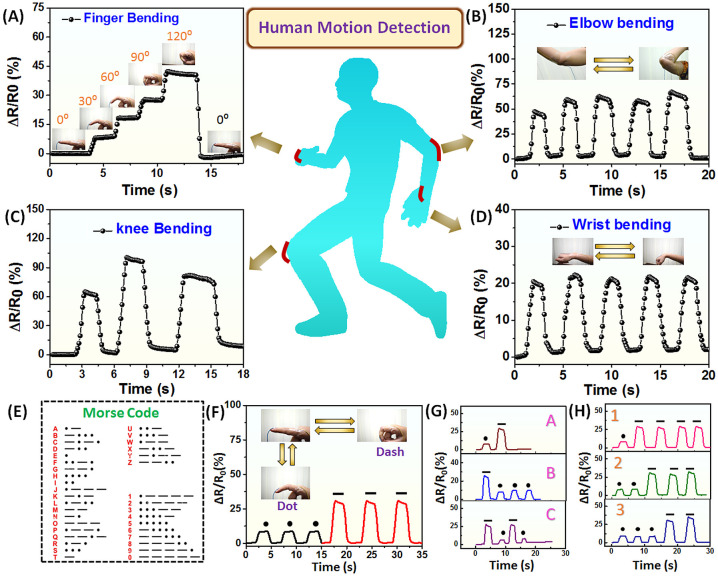
The AM_20_-Ca^2+^ hydrogel-based strain sensor for human motion detection. The relative resistance change in response to the bending of (A) finger, (B) elbow, (C) knee, and (D) wrist. (E) Morse code for representing the English alphabet and numerical numbers. (F) Representation of Morse code through the relative resistance change by finger bending. Relative resistance change corresponding to the bending from 0° to 30° is representing the dot, and relative resistance change corresponding to the bending from 0° to 90° is representing the dashed line. (G) Representing Morse code for the English alphabet (A, B, C) by the relative resistance change of finger bending. (H) Representing Morse code for numerical numbers (1, 2, 3) by the relative resistance change of finger bending.

The AM_20_-Ca^2+^ hydrogel-based strain sensor, owing to reasonable ionic conductivity, flexibility, good strain sensitivity, excellent adhesiveness, and effective electrical healability, was attached onto various parts of the human body (such as, finger, elbow, knee, and wrist) for movement monitoring (Figure 10A–D). Moreover, because of the high mechanical recovery and consistent electrical response of this hydrogel material, it can be employed to repeatedly detect a variety of movements occurring in the human body. As illustrated in Figure 10A, the hydrogel-based strain sensor device was attached to an index finger. Throughout the gradual bending of the finger (from 0° to 30°, 60°, 90°, 120°), the relative resistance change gradually increases due to stretching of hydrogels, with minimal fluctuation in resistance observed at each bending state. The sensor is capable of restoring its initial resistance when the finger becomes straight (0°). Further, upon attachment of the hydrogel strain sensor to the elbow, it efficiently detects every bending and straightening motion, showcasing periodic resistance changes in response to the consistent bending actions (Figure 10B). Additionally, when attached to the knee, the sensor accurately changes its resistance, in line with each leg flexion, matching the frequency of movement (Figure 10C). The bending of the wrist was perfectly detected by attaching the hydrogel sensor onto it (Figure 10D). Upon bending the wrist, the hydrogel sensor showed an increase in relative resistance and again after straightening it recovers its relative resistance change. Assessing the cytotoxicity of these hydrogel materials used for strain-sensing applications is essential because of their possible contact with the skin. To investigate this aspect, live/dead assay (Calcein-AM and MTT) was performed after culture of the mouse L929 fibroblast cells with AM_20_-Ca^2+^ hydrogels of different concentrations. The results of the MTT assay showed that the percentage of cell viability remained above 75% across the different hydrogel concentrations (Figure S6). The fluorescence microscopy images from the Calcein-AM assay also indicated that most of the cells were alive on culture with different concentrations of the AM_20_-Ca^2+^ hydrogels (Figure S7). These results suggest that the hydrogel is noncytotoxic and biocompatible. The L929 mouse fibroblast cells have high cytotoxic response, similar to human fibroblast cells.^53−56^ Therefore, these cells have been widely employed for cytotoxicity studies of different synthetic materials. Hence, based on the results of the present studies, it is expected that our hydrogel will be noncytotoxic toward the human skin.

Hydrogel strain sensors have the capability to not only monitor human limb movement but also transmit information, thereby enabling opportunities for information encryption/decryption and enhancing information accessibility for individuals with speech disabilities. Morse code is an internationally recognized silent language used to convey information, employing “dots” and “short lines” to represent various English letters and numbers (Figure 10E). When the AM_20_-Ca^2+^ hydrogel sensor was attached on the finger to maintain a bending from 0 to 30° angle, the corresponding relative resistance change was represented as dots. When the finger wasbent from 0 to 90° angle, the corresponding resistance change was higher, which was denoted as dashed lines (Figure 10F). Utilizing this strategy, through finger movement, different English letters (A,B,C) and numbers (1,2,3) have been represented ((Figure 10G,H)). The consistent and stable changes in current signals confirmed the reliability of hydrogel sensors. This supports their potential use in new areas like encrypting/decrypting information, delivering messages, and facilitating communication for people who are deaf or mute.

### Flexible Supercapacitor Performance

**Figure 11:**
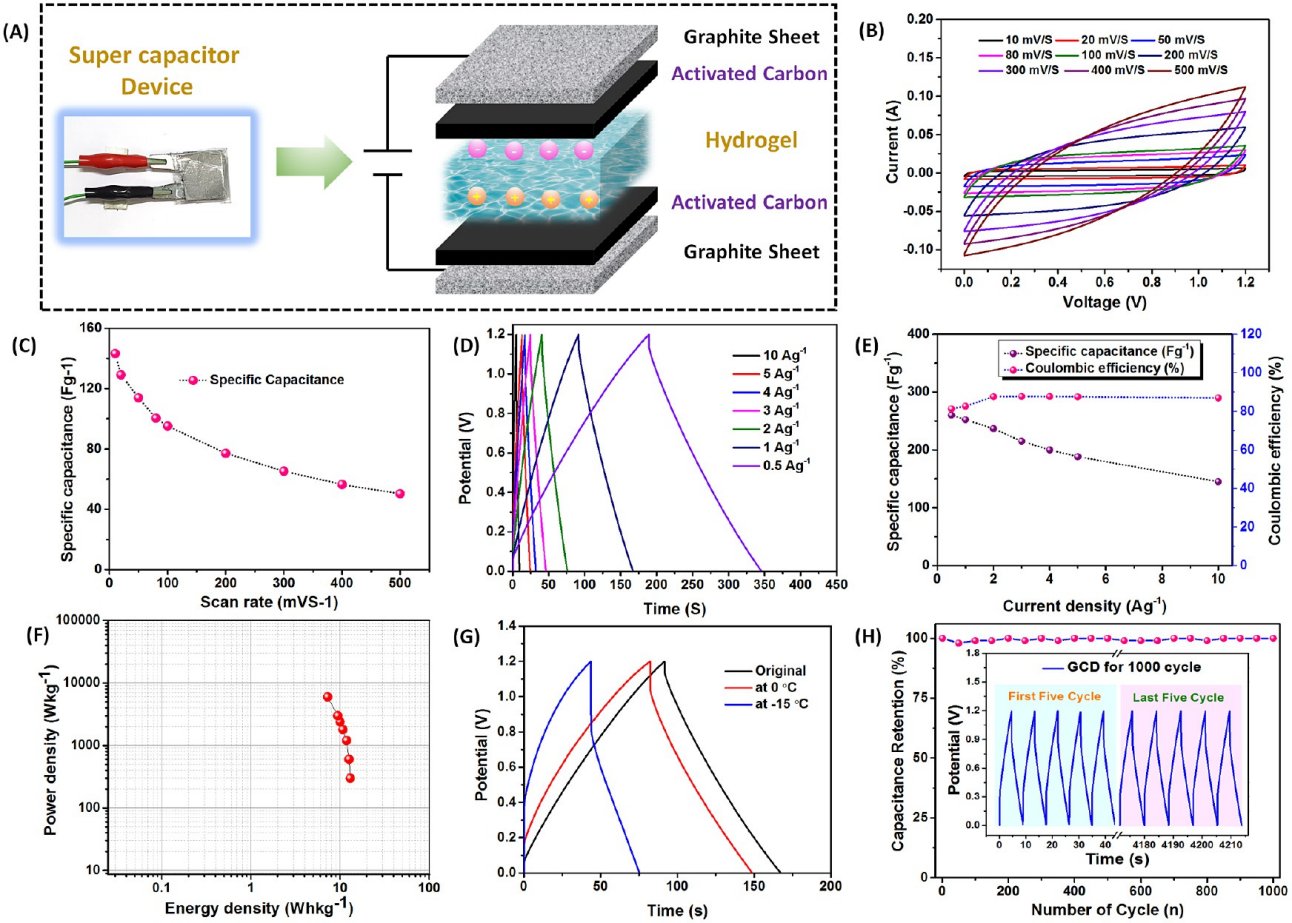
(A) Schematic of the solid electrolyte-based supercapacitor device and its charging–discharging mechanism. (B) Cyclic voltammetry (CV) curve. (C) Variation of specific capacitance with scan rate. (D) Galvanostatic charge–discharge (GCD) profile. (E) Specific capacitance and Coulombic efficiency as a function of current density. (F) Power density vs current density plot at different current densities. (G) GCD profile at different temperatures. (H) Capacitance retention during 1000 GCD cycles.

The AM_20_-Ca^2+^ hydrogel was employed as a solid electrolyte in a flexible supercapacitor device. Ionic conductivity, a porous microstructure, and the ability to adhere to the electrode surface make the AM_20_-Ca^2+^ hydrogel a suitable choice for solid electrolyte applications. Simultaneously, it is also highly stretchable and strong, which should be an advantage to fabricate a flexible supercapacitor device. This hydrogel can act as both the electrolyte and separator in the device, which helps in preventing liquid leakage and eliminates the need for external additives.^57,58^ Its antifreezing, antidehydration, and self-healing properties also make the device more durable. To construct the supercapacitor device, the AM_20_-Ca^2+^ hydrogel was placed between two carbon-coated graphite electrodes (Figure 11A). Even though the hydrogel has antidehydration properties, the whole device was still covered with insulating tape to prevent any water loss. To comprehensively evaluate the electrochemical characteristics of the supercapacitor device, a series of experiments, including cyclic voltammetry (CV), galvanostatic charge–discharge (GCD), and electrochemical impedance spectroscopy (EIS), were conducted. These investigations provide insights into the capacitive behavior, energy storage capabilities, and impedance properties essential for understanding the performance of the device.

The CV experiment was performed by varying the scan rate from 10 to 500 mV s^–1^ in the potential range of 0–1.2 V (Figure 11B). The data exhibit the symmetrical quasi-rectangular CV curve, denoting electrochemical double layer capacitance (EDLC) behavior for the supercapacitor.^59,60^ As the scan rates were progressively increased, the hydrogel-based supercapacitor exhibited a consistent curve with a notable enhancement in the CV loop area, attributed to reversibility and reproducibility. The specific capacitance values obtained from the CV analysis at various scan rates are shown in Figure 11C. These results demonstrate that increased scan rates do not provide sufficient time for the ions adsorption/desorption on the electrode surface resulting in decreased specific capacitance. It was observed that even up to 1.5 V, this device exhibits a consistent CV profile, demonstrating its efficiency for functioning within a wide potential range (Figure S8A). For the same current density, the specific capacitance also gradually increased with an increasing potential window, as calculated from the GCD data that were obtained at different potential windows at a current density of 1 Ag^1–^ (Figure S8B,C).

GCD tests were performed to investigate the charge–discharge mechanism and precisely determine the specific capacitance by varying the current density. Figure 11D shows that within the potential window of 0–1.2 V, the GCD curves exhibited isosceles triangles with minimum IR drop under current densities of 0.5–10 Ag^–1^, indicating reversible charging and discharging behavior between the electrode and the electrolyte.^61^ The specific capacitance data calculated from the GCD data at different current densities are shown in Figure 11E. It was observed that the specific capacitance increased with decreasing current density and showed a maximum value (∼260 F g^–1^) at 0.5 Ag^–1^. The Coulombic efficiency is measured to be in the range of 85–90% at current densities between 2 and 10 Ag^–1^. A comparison with the different hydrogel electrolyte-based supercapacitor devices (Table S3) indicates the potentiality of the device reported in our work.^18,26,62−65^ As discussed earlier, this hydrogel-based supercapacitor device exhibited stable electrochemical performance over a large potential window and the CV curve showed that even at 1.5 V, this device showed EDLC behavior (Figure S8A). Figure 11F shows the Ragone plot showing the energy density and power density of the AM_20_-Ca^2+^ hydrogel electrolyte-based supercapacitor device. This AM_20_-Ca^2+^ hydrogel-based supercapacitor exhibited energy density up to ∼13 Wh kg^–1^ and maximum power density of this device was 6000 W kg^–1^, which was higher than the other gel electrolyte-based supercapacitors.^18,63−65^ Due to the remarkable ability of the AM_20_-Ca^2+^ gel electrolyte to retain both its flexibility and conductivity at subzero temperatures, the performance of the supercapacitor device was also investigated at subzero temperatures. The device was able to maintain the same EDLC behavior in the CV curve even at −15 °C (Figure S9). GCD experiments were conducted at 0 and −15 °C at a current density of 1 Ag^–1^(Figure 11G). A maximum specific capacitance of 105.3 F/g at a current density of 1 Ag^–1^ could be achieved even at −15 °C, which is ∼42% of the specific capacitance measured at room temperature. These results suggest that the AM_20_-Ca^2+^ hydrogel can be a suitable gel electrolyte for supercapacitor devices at both ambient atmosphere and subzero temperatures. The cyclic stability of the supercapacitor device was tested by using 1000 GCD charge–discharge cycles at a current density of 1 Ag^–1^ at ambient temperature. The results showed that ∼99% capacity was retained even after 1000 cycles (Figure 11H).

Since the hydrogel-based solid electrolyte is mechanically robust and flexible and has good self-recovery properties, the electrochemical performance of the supercapacitor device was checked under different mechanical deformations like putting different compressive loads on top of the device or bending the supercapacitor device. First, different compressive loads were applied to the device and GCD experiments were conducted (Figure S10A). Upon applying various loads: 125, 250, and 500 g cm^–1^, the specific capacitance slightly increased and on removing the loads, the device recovered its original capacitance (Figure S10B). On applying different loads, the consequent compression of the electrolyte results in the reduction of electrical resistance. Furthermore, the flexible supercapacitor device was bent up to a certain angle (>90°) and the GCD performance was checked (Figure S10C). The result showed that the specific capacitance decreased slightly on bending the device (Figure S10D). This can be attributed to the stretching of the electrolyte within the hydrogel during bending, which enhanced the electrical resistance and decreased the specific capacitance of the device. Again after straightening, the device was able to recover the original specific capacitance due to the good self-recovery property of the hydrogel.

Environmental stability is also important for enhancing the lifetime of gel-based supercapacitor devices. Since this gel showed excellent antidrying properties, after 30 days also, it showed similar CV and GCD curve with a negligible loss (∼8%) in specific capacitance (Figure S11). In brief, this hydrogel-based supercapacitor device was constructed with a fundamentally simple concept, yet its impressive performance across a wide temperature range and long-term durability have made it a promising material for hydrogel-based supercapacitor devices.

### EMI Shielding Performance

**Figure 12:**
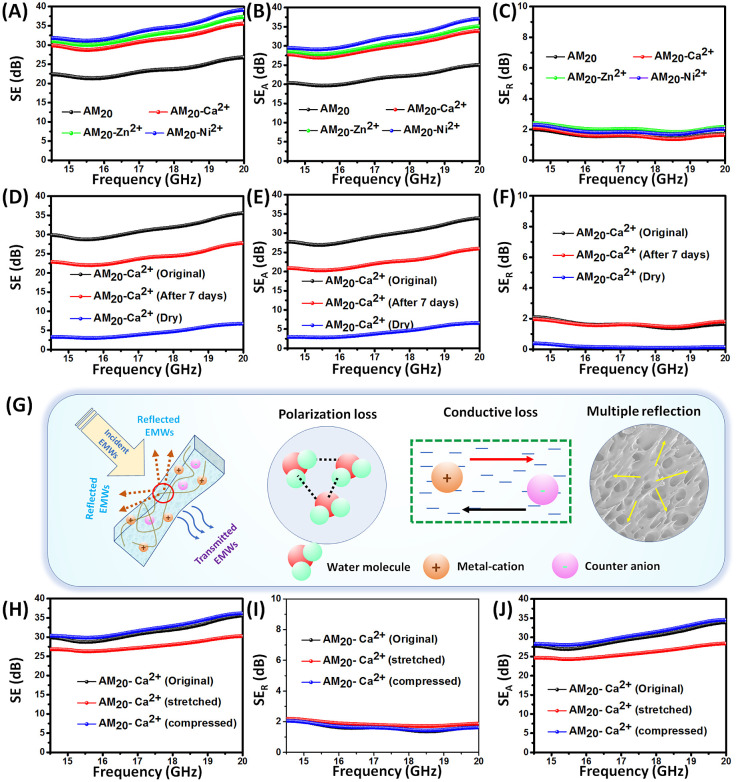
(A) SE, (B) SE_A_, (C) SE_R_ of AM_20_ hydrogels before and after incorporation of different metal ions (Ca^2+^, Ni^2+^, and Zn^2+^); variation of (D) SE, (E) SE_A_, (F) SE_R_ of AM_20_-Ca^2+^ hydrogels at different water content; (G) the EMI shielding mechanism of metal ion cross-linked hydrogels mainly includes factors such as polarization loss of water molecule, conductive loss due to migration of charge species, and scattering from porous network; variations of (H) SE, (I) SE_R_, (J) SE_A_ of AM_20_-Ca^2+^ hydrogels after stretching and pressing. Here, SE, SE_R_, and SE_A_ represent the total shielding efficiency, reflection-based shielding efficiency, and absorption-based shielding efficiency

The AM_20_-Ca^2+^hydrogel sample was utilized to investigate its capability to shield against electromagnetic interference (EMI). To study the EMI shielding effectiveness, a 1.1-mm-thick AM_20_-Ca^2+^ hydrogel was subjected to a vector network analyzer over a frequency range of 14.5–20 GHz. The total shielding efficiency (SE) was measured using various scattering parameters (S_11_, S_12_, S_21_, S_22_) and equation 7 of the Supporting Information, which measures the attenuation of electromagnetic waves passing through the hydrogel.^66^ At a frequency of 20 GHz, the AM_20_-Ca^2+^ hydrogel displayed a total shielding efficiency (SE) of ∼35 dB (Figure 12A), which is far exceeding the industrial standard of 20 dB.^67−69^ This value also surpassed that of many other conducting filler-based elastomeric and hydrogel materials used for electromagnetic interference (EMI) shielding applications^66,70−78,79−86^ (Table S4). This exceptional EMI shielding ability of the AM_20_-Ca^2+^ hydrogel may be attributed to three key factors: high water content (∼74%), a porous network, and adequate ionic conductivity. Water is a polar molecule, and under the influence of electromagnetic fields it can polarize and form hydrogen bonds, leading to a disruption of network structures.^87^ This consequential polarization relaxation loss of water in the gigahertz (GHz) and terahertz (THz) bands significantly diminishes the energy of electromagnetic wave (EMW) radiation.^88−91^ Second, high ionic conductivity of the hydrogel helps to increase the shielding efficiencies through the absorption and reflection losses. Additionally, the hydrogel is composed of various chemical and physical cross-linking bonds, resulting in a porous structure (revealed form FESEM analysis) that effectively reflects and absorbs electromagnetic radiation (Figure 12B,C).^92,93^ The presence of multiple ionic components, especially metal ions like Ca^2+^, gives the hydrogel a moderate to high ionic conductivity, which contributes to absorption loss.^73,94,95^ The function of metal ions and their impact on ionic conductivity became evident when comparing the shielding efficiency of the hydrogel to that without metal ions (AM_20_ hydrogels). The AM_20_ hydrogel showed significantly lower shielding efficiency (SE = 26.78 dB). The absence of metal ions resulted in a sharp decrease in conductivity, from 19.5 to 1.4 mS cm^–1^, which in turn reduced the shielding efficiency of the hydrogel. However, when Ni^2+^ and Zn^2+^ ions were incorporated instead of Ca^2+^ ions, the ionic conductivity was slightly enhanced (ionic conductivity for AM_20_-Ni^2+^ and AM_20_-Zn^2+^ hydrogels are 21.7 and 20.1 mS cm^–1^, respectively), leading to an increase in the SE for the AM_20_-Ni^2+^ (SE = 39.14 dB) and AM_20_-Zn^2+^ (SE = 37.30 dB) hydrogels.

The total EMI shielding efficiency (SE) comprises two mechanisms: reflection (SE_R_) and absorption (SE_A_), which are attributed to mobile charge carriers and electric dipoles, respectively. Therefore, individual calculations of SE_A_ and SE_R_ can reveal the actual shielding mechanism of the hydrogel. Separate calculations of SE_A_ and SE_R_ showed that at 20 GHz frequency, SE_A_ for the AM_20_ hydrogel was approximately 25 dB, which increased to around 33.9, 35.1, and 37.1 dB with the incorporation of Ca^2+^, Ni^2+^, and Zn^2+^ ions, respectively. However, there was no significant increase in SE_R_ (<2.2 dB), indicating that the shielding mechanism for these metal ion cross-linked hydrogels is mostly absorption dominated. Since the value of SE_R_ < 3 dB and SE_A_ > 30 dB, green index (g_s_) >1, these hydrogels are in the category of most desirable green EMI shielding materials because their reflected radiation will not have any adverse effect toward the other external device as well as human health.^96,97^ Although the shielding efficiencies of the AM_20_-Ca^2+^ hydrogel were slightly lower than those of the AM_20_-Ni^2+^ and AM_20_-Zn^2+^ hydrogels, considering its mechanical and other functional properties (like self-healing, antifreezing, and antidrying), the AM_20_-Ca^2+^ hydrogel was chosen for further analysis.

Apart from the metal ions, total water content inside the hydrogel plays a critical role in enhancing the electromagnetic shielding performance of the hydrogel. The presence of water molecules within the hydrogel’s network forms a network of hydrogen bonds and undergoes polarization when subjected to a magnetic field. Thus, changes in the water content directly affect the electromagnetic interference shielding efficiency (EMI SE). The role of water was observed when the shielding efficiencies of the AM_20_-Ca^2+^ hydrogel was compared with its dry state (Figure 12D–F). It was observed that after completely drying, the AM_20_-Ca^2+^ hydrogel provided total shielding efficiency of 6.6 dB, which is ∼6 times lower than its gel state. This sharp reduction in shielding efficiencies was attributed to the absence of polarization loss. As a result, there were sharp declines in SE_A_ and SE_R_.

The traditional hydrogels are prone to rapid drying under working conditions, resulting in a significant loss of EMI SE. Since the AM_20_-Ca^2+^ hydrogel exhibits antidehydration properties, this material is able to maintain its EMI SE over longer periods of time. As shown in Figure 12D, the EMI SE of the hydrogel with varying water content gradually decreased over time when exposed to the open air, reaching approximately 26 dB after 7 days. Although the AM_20_-Ca^2+^ hydrogel possesses antidehydration properties, the decrease in EMI SE is undesirable. Therefore, the AM_20_-Ca^2+^ hydrogel was sandwiched between two VHB tapes, and even after 30 days, the EMI SE remained almost constant at approximately 33.7 dB (Figure S12).

The EMI shielding performance of the AM_20_-Ca^2+^ hydrogel was also affected by the mechanical deformation of the hydrogel. To measure this, the AM_20_-Ca^2+^ hydrogel was stretched to 100% and the corresponding EMI shielding effectiveness (SE) was measured. It was noted that stretching the hydrogel resulted in a ∼ 42% decrease in SE (20.1 dB). When the hydrogel was pressed gently by a finger and the experiment was repeated, a small (∼3%) enhancement (i.e., 36.3 dB) in the shielding efficiency was measured. This finding is consistent with the previous report.^73,98,99^ The decrease in shielding efficiency during stretching may be attributed to reduction of thickness as well as increase in electrical resistance.^98^ Likewise, the shielding efficiency increased on pressing the hydrogel because of the reduction in resistance. As the hydrogel mainly exhibited absorbance-based shielding, both stretching and pressing had a greater effect on the absorbance shielding (SE_A_). However, stretching did result in a small increase in reflection-based shielding (SE_R_), possibly due to a more aligned orientation of the polymer chains during the stretching process.

The conventional hydrogels lose their ability to shield against electromagnetic interference at subzero temperature because of the onset of freezing of water inside the hydrogel. However, unlike conventional hydrogels, the AM_20_-Ca^2+^ hydrogel possesses antifreezing properties that should help maintain its EMI shielding ability even at subzero temperatures. To check this, the VHB tape encapsulated hydrogel was stored at −15 °C for 12 h, after which its shielding efficiency was measured. Remarkably, the hydrogel still maintained its flexibility and achieved a total shielding efficiency of ∼24 dB, even at −15 °C (Figure S13A). The antifreezing property of the hydrogel allowed continued migration of ions and contributed to excellent EMI shielding even in freezing conditions. However, the overall shielding efficiency was lower at subzero temperatures because of the decrease in ionic conductivity.

Finally, the practical application of the EMI shielding performance of this hydrogel has been demonstrated by blocking the wifi signal from the cell phone (Figure S13B). For this, first a 4G cell phone was wrapped with aluminum foil. Since the aluminum foil is a well-known EMI blocking element, it blocks the wifi comes out. Next the phone was covered with aluminum foil, keeping one cavity for the signal transportation. Through this cavity, signal transportation occurs; therefore, wifi signal still works. Further that cavity was blocked by this transparent hydrogel, and it was observed that the wifi signal was blocked. Thus, this hydrogel proves its potential in effectively shielding the electromagnetic radiation.

## Conclusions

In summary, we have successfully synthesized dynamic metal ion cross-linked adhesive and self-healing hydrogels through thermal copolymerization of acrylamide and maleic acid monomers and in situ incorporation of metal ions. The hydrogel relies on a dual cross-linked approach, featuring a small quantity of chemical cross-links and a high amount of physical cross-linking involving metal–ligand interactions and H-bonding. These hydrogels possess remarkable mechanical strength, flexibility, self-healing capabilities, and ionic conductivity, making them ideal for use in various fields such as strain sensing, energy storage, and electromagnetic shielding. The incorporation of Ca^2+^-dicarboxylate dynamic metal–ligand cross-links in combination with low density chemical cross-links has resulted in a hydrogel with excellent mechanical properties and diverse functionalities. With its simple synthesis route and multifunctional properties, this hydrogel has the potential to revolutionize the field of materials science and open up new avenues for research and development. The continuous advancements and exploration in this field will undoubtedly lead to further improvements and applications of dynamic metal-coordinated adhesive and self-healable hydrogels, making them invaluable additions to the arsenal of modern materials.

## Supporting Information Available

Investigation of conductive properties (MP4)
Synthesis of the hydrogels and characterization procedures (mechanical properties, differential scanning calorimetry, microscopy, XPS spectroscopy, FTIR, resistive sensing, electrochemical measurements, EMI shielding efficiency measurements), additional experimental results (PDF)
