# Supplementary material for: Dynamic Metal-Coordinated Adhesive and Self-Healable Antifreezing Hydrogels for Strain Sensing, Flexible Supercapacitors, and EMI Shielding Applications
Source: ACS Omega. 2024 Jul 21;9(30):33204–23. doi: 10.1021/acsomega.4c04851 (PMC11292641; doi:10.1021/acsomega.4c04851)
Supplement: Supplementary file 2 — ao4c04851_si_002.pdf [file ao4c04851_si_002.pdf]

Supporting Information

Dynamic Metal-Coordinated Adhesive and Self-Healable Antifreezing Hydrogels for Strain sensing, Flexible Supercapacitors and EMI Shielding Applications

Ashis Ghosh[1], Sudhir Kumar[1], Prem Pal Singh[1], Suvendu Nandi[2], Mahitosh Mandal[2], Debabrata Pradhan[1], Bhanu Bhusan Khatua[1], Rajat Kumar Das[1]*

[1]Materials Science Centre, Indian Institute of Technology Kharagpur, Kharagpur, India

[2]School of Medical Science and Technology, Indian Institute of Technology Kharagpur, Kharagpur, India

Correspondence

Rajat Kumar Das, Materials Science Centre, Indian Institute of Technology Kharagpur, Kharagpur, West Bengal 721302, India.

Email:rajat@matsc.iitkgp.ac.in

Materials:

Acrylamide (AM)[SRL], Maleic acid (MA) [Loba Chemie], Methylene bis acrylamide (MBAA)[Sigma Aldrich], Ammonium persulfate (APS)[Sigma Aldrich], Calcium Chloride [Merck], Ferric nitrate nonahydrate [SRL], Ferrous sulfate heptahydrate [Merck], Cupric sulfate pentahydrate [Loba Chemie], Zinc acetate dihydrate [Loba Chemie], Nickel chloride hexahydrate [SRL] were used as a received. All the experiments were done using Deionized (DI) water.

Synthesis of AM_x-M^{n+} hydrogel:

Dynamic metal-ligand interaction based AM_x-M^{n+} hydrogel was synthesized through thermal initiated co-polymerization method. The synthesis process of hydrogel was illustrated in Scheme S1. Total monomer concentration was fixed at 25 wt.% and two hydrophilic monomer acrylamide and maleic acid were taken at mentioned ratio (in **Table S1**). In the procedure of gel formation, a mixture containing acrylamide, maleic acid and MBAA (0.05 wt% of total monomer) were taken in a test tube and stirred for 30 min for homogeneous mixing. Then metal ion-based salt was added to this solution at appropriate molar ratio w.r.t. maleic acid and again allowed to stir for 30 min. After nitrogen bubbling, initiator APS (1 wt% of total monomer) was added to it and subsequently transferred into a petridish. Next it was kept in hot air oven at 60 °C for 12 h.

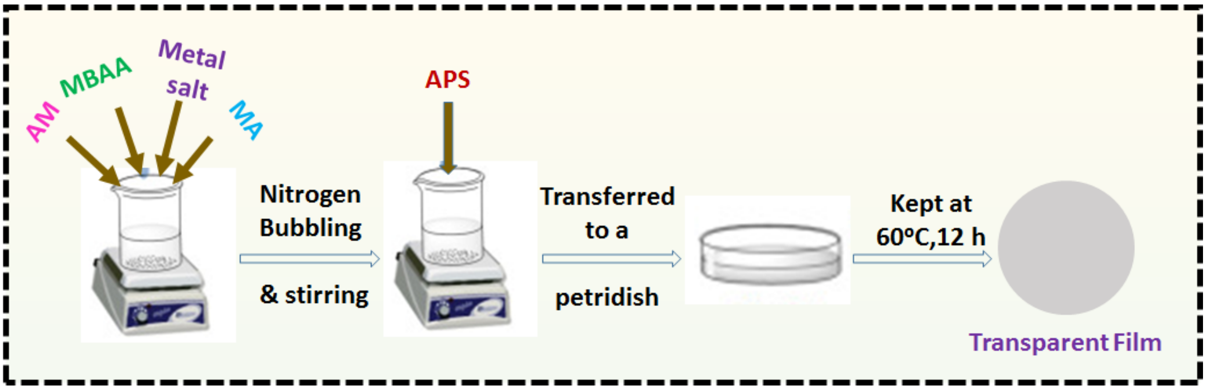

Scheme S1. Synthesis of AM_x-M^{n+} hydrogel and cross-linking with metal ions.

Table S1. Compositions of AM_x-M^{n+} hydrogel.

Hydrogel	Acrylamide (AM) (mg)	Maleic acid(MA) (mg)	MBAA (mg)	APS (mg)	Metal ion: carboxylate molar ratio	Water (mL)	Remarks
AM_{10}	450	50	0.5	5	-	2	Gel
AM_{10}-Ca^{2+}	450	50	0.5	5	1:2	2	Gel
AM_{10}-Ni^{2+}	450	50	0.5	5	1:2	2	Gel
AM_{10}-Zn^{2+}	450	50	0.5	5	1:2	2	Gel
AM_{15}-Ca^{2+}	425	75	0.5	5	1:2	2	Gel
AM_{20}-Ca^{2+}	400	100	0.5	5	1:2	2	Gel
AM_{25}-Ca^{2+}	375	125	0.5	5	1:2	2	Gel
AM_{10}-Fe^{2+}	450	50	0.5	5	1:2	2	No Gel
AM_{10}-Fe^{3+}	450	50	0.5	5	1:3	2	No Gel
AM_{10}-Cu^{2+}	450	50	0.5	5	1:2	2	No Gel

Mechanical testing:

All mechanical properties of the hydrogels were tested in Zwick/Rowell Z050 Universal Testing Machine at room temperature. All samples were coated with a thin silicone oil layer to prevent water removal prior to every test. The measurements were performed on rectangular shaped strips having thickness ~1.2mm, width ~5mm and length ~12 mm. Tensile experiments were done by using 100 N load cell at 25 ºC and at the crosshead speed of 100 mm/min. The engineering stresses were calculated using the following formula: = F/A_0, where, F is the applied force and A_0 is the initial cross-sectional area. The strain% was calculated as $(L_i-L_0)*100/L_0$, where L_i is the final length and L_0 is the initial length. Toughness was calculated from the area under the stress strain curve (calculated by using Origin 9.0 software). Elastic modulus was calculated from the slope of stress strain curve in the strain range of 5-20%. The tensile cyclic loading and unloading test was performed at a strain rate 100 mm/min. The energy dissipations were calculated from the area under the loading and unloading curve. The analysis was done by origin software. Successive loading and unloading tests were performed for 10 cycles up to 100% strain with a strain rate of 100 mm/min.

Compression Test:

Compression tests were performed using 50 kN load cell with a crosshead speed 3 mm/min. Samples were made in cylindrical shape with a diameter 14 mm and height 20 mm. Compressive stress was measured by dividing force (F) by the original cross-sectional area. Compression strain was taken as the ratio of deformed height and original height.

Fracture Test:

Fracture energy of AM_{10}-Ca^{2+} hydrogel was determined by employing Rivlin and Thomas's method[1]. A rectangular shaped hydrogel was taken with width 20 mm, thickness 0.75 mm and stick to polystyrene sheet with glue. The distance between two grips was fixed at 6 mm. Both notched (40% cut along width) and unnotched samples were subjected to tensile test three times. From notched sample, a critical length(Lc) is obtained, which denotes the starting of crack propagation. From unnotched sample a tensile stress-strain plot was drawn and from this plot area up to the critical length was calculated, which is the applied force U(Lc).

Fracture energy of the sample was calculated from the following equation,

Γ= U(Lc)/a_0b_0, a_0= width of sample, b_0 =thickness of sample

Differential Scanning Calorimetry (DSC):

The DSC experiment was carried out in a NETZCH Maia DSC 200 F3 instrument at a cooling rate of 5 ℃ min^{-1}.The heating curve was taken for analysis.

Dynamic mechanical Analysis (DMA):

DMA analysis was performed by subjecting a cylindrical shaped (~10 mm diameter and ~5mm height) hydrogel sample to NETZSCH DMA-242 E Artemis instrument in compression mode from 40 °C to – 40 °C at a cooling rate of 5 °C min^{-1} and frequency of 1 Hz.

Field emission scanning electron microscope (FESEM) analysis:

Surface morphology of all hydrogels were studied by Zeiss Gemini500 or Zeiss VP 300 field emission scanning electron microscope, operating at 5 kV. Hydrogel were taken as freeze-dried thin film samples. Samples were gold coated for 5 min before analysis. The mapping of metal ions using Energy Dispersive X-ray Spectroscopy (EDS) was conducted at a magnification of 5kX and a scanning duration of 3 minutes.

XPS spectroscopy:

X-ray photoelectron spectroscopy (XPS) measurement of the freeze dried $AM_{10}B_{10}$-Fe^{3+} hydrogel film was studied by with a PHI 5000 VersaProbe-II (ULVAC, PHI, Inc.) spectrometer, equipped with a micro-focused (100 µm, 25 W, 15 kV) monochromatic Al-K_{α} X-ray source with an energy of 1486.6 eV and base pressure ~2.6×10^{-6} Pascal. The binding energy was corrected taking the C 1s peak at 284.5 eV. The XPS peaks were fitted using XPSPEAK41 software.

FTIR analysis:

The ATR-FTIR spectra of the freeze-dried hydrogel film were obtained using a Perkin Elmer spectrum-II spectrometer and analysed using the Origin 9 software.

Ionic conductivity measurements:

Ionic conductivity of the hydrogel was measured by using high performance AC impedance analyser (Win Delta Novotherm α). Experiments were carried out in the frequency range of 10 Hz to 10 MHz at an electrical potential difference of 1V. A circular shaped sample (diameter: 13mm, and thickness: 1.1 mm) was used for this experiment. The Ionic conductivity (σ, mS/cm) was calculated from this following formula:

$$\sigma = \frac{l}{R * A} \qquad (1)$$

Where l =the distance between two electrodes, A= Effective area (A= $\Pi * D^2/4$, where D=diameter of the sample), R= Resistance Calculated from the Nyquist plots.

Resistive sensing:

The resistive sensing application of the $AM_{10}B_{10}$-Fe^{3+} hydrogel (20 mm x 4.5 mm x 1.1 mm) was performed using a Hioki LCR meter. The gel sample was subjected to different strain% (0%, 50%, 100% and 150 %) and relative resistance change was calculated accordingly. Resistance change was measured at a fixed frequency of 10 kHz. The relative resistance change was calculated with this formula

$$\Delta R = \frac{R_i - R_0}{R_0} \qquad (2)$$

where the R_0 and R_i were the resistance at 0% strain and different strains respectively. The sensitivity of strain sensor was measured in terms of gauge factor which is defined as %ΔR / Strain%.

Self-healing Test:

The macroscopic self-healing ability of AM_{20}-Ca^{2+} hydrogel was investigated. Initially, a rectangular AM_{20}-Ca^{2+} hydrogel was divided into two half and then re-join and sealed together in a zipper packet for varying durations. The healed hydrogel was then tested for tensile strength and observed for visual healing after stretching, bending or twisting. The effectiveness of the hydrogel's self-healing was assessed by measuring the recovery of mechanical properties at different intervals.

Electrochemical Measurements:

The performance of fabricated supercapacitor devices has been demonstrated using a CHI 760D electrochemical workstation (CH Instrument, Inc.,USA). The device was formed by sandwiching the hydrogel between two activated carbon coated graphite sheets. The performance of supercapacitor device has been measured by doing cyclic voltammetry (CV) galvanostatic charge-discharge (GCD) and electrochemical impedance spectroscopy (EIS). Specific Capacitance (C_s, Fg^{-1}) was calculated using the following formula[2] with the help of galvanostatic charge-discharge (GCD) profile:

$$C_s = \frac{4 * I * \Delta t}{m * \Delta V} \quad (3)$$

Energy Density (E, W h Kg^{-1}) and power density (P, W Kg^{-1}) were calculated using these following equations[3]:

$$E = \frac{C_s * \Delta V^2}{2 * 4 * 3.6} \quad (4)$$

$$P = \frac{3600E}{\Delta t} \quad (5)$$

Where, I (A) and Δt (s) is discharge current and time respectively, ΔV (V) is potential window and 'm' is total mass of active materials, deposited in two electrodes.

To investigate the dependence of the specific capacitance on the scan rate, the specific capacitance was also calculated utilizing the data from the CV plots obtained at different scan rates, using the following equation[4]:

$$C_s = \frac{A}{\Delta V * \upsilon * m} \quad (6)$$

where A is the integral area of the cyclic voltammetric curve, ΔV is the potential window, υ is the scan rate and m is the mass of active materials in the electrode.

EMI shielding efficiency measurements:

The EMI shielding effectiveness (SE) of a 1.5 mm-thick hydrogel was analyzed using a vector network analyzer (VNA) over a frequency range of 14.5–20 GHz. Various scattering parameters (S11, S12, S21, S22) were utilized to measure the SE according to the equation 9, which determines the attenuation of the electromagnetic waves passing through the hydrogel The total EMI SE is evaluated by scattering parameters obtained from VNA using the following equations:

$$SE = 10\log_{10}\left(\frac{1}{|S_{12}|^2}\right) = 10\log_{10}\left(\frac{1}{|S_{21}|^2}\right) \quad (7)$$

$$SE_R = 10\log_{10}\left(\frac{1}{1-|S_{11}|^2}\right) \quad (8)$$

$$SE_A = 10\log_{10}\left(\frac{1-|S_{11}|^2}{|S_{12}|^2}\right) \quad (9)$$

where, SE is total EMI SE, SE_R is EMI SE due to reflection, SE_A is EMI SE due to absorption, S_{12} is reverse transmission coefficient, S_{21} is forward transmission coefficient, S_{11} is forward reflection coefficient, and S_{22} is reverse reflection coefficient. All scattering parameters were measured in the region of extended K_u-band (14.5–20 GHz). The overall shielding effect is a combination of contributions from absorption, reflection, and multiple reflections. Therefore, the resulting SE can be expressed as,

$$SE = SE_A + SE_R + SE_M \quad (10)$$

However, the EMI SE due to multiple reflections (SE_M) is recognized to be negligible in real-world applications when the shielding material thickness is greater than the skin depth (the distance required to attenuate the EM radiation by 1/e times, or 37% of the original strength)

or when the EMI SE_T is more than 15 dB [1]. Therefore, neglecting the contributions of SE_M, the resulting SE can be expressed as,

$$SE \approx SE_A + SE_R$$

MTT assay:

Cytotoxicity testing of the AM_{20}-Ca^{2+} hydrogel was performed on the mouse L929 fibroblast cell line using an MTT assay. Once the cells were fully confluent, they were plated in 96-well plates (8×10^3 cells per well) and incubated for 24 h. The cells were then treated with the AM_{20}-Ca^{2+} hydrogel at different concentrations. After incubation for 48 h, the cells were assessed using the MTT assay protocol (Sigma-Aldrich, USA). The optical density was measured at 595 nm, and the results were analyzed. The absorbance of the samples was measured at 595 nm using a microplate reader (BioRad, iMark, Japan).

Live/dead staining (Calcein-AM):

Calcein AM (acquired from Thermo Fisher Scientific), USA and Propidium Iodide (acquired from Sigma Aldrich) staining were used to determine the viability of the cells by identifying live and dead cells by their respective fluorescence emissions (live cells were stained green by Calcein AM, while dead cells were stained red by Propidium Iodide). Mouse L929 fibroblast cells were seeded at a viable concentration of 5.8×10^6 cells/mL. The AM_{20}-Ca^{2+} hydrogel was added to serum-free media (DMEM) at different concentrations (100, 500, and 1000 μg/ml), and cells were treated accordingly. After 24 h of incubation, the cells were stained and images were captured using fluorescence microscopy (Nikon, ECLIPSE Ts2 Inverted Microscope) allowing for the assessment of cell viability.

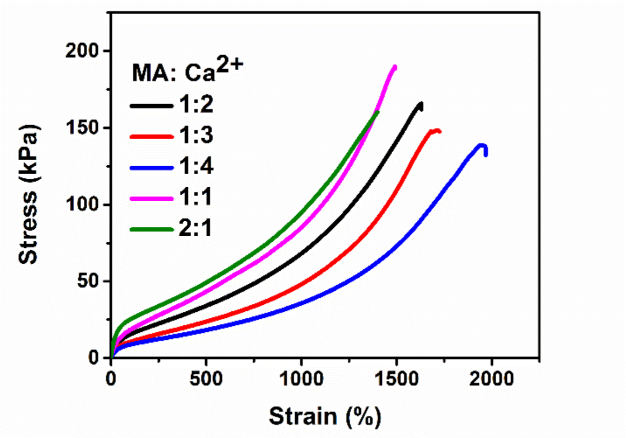

Figure S1. Tensile stress-strain experiments with AM_{10}-Ca^{2+} hydrogels with different molar ratios of maleic acid (MA)/Ca^{2+}.

Table S2. Tensile strength of AM_{10}-Ca^{2+} hydrogel with different metal ion/ligand molar ratios.

Hydrogel	Maleic acid:Ca^{2+} molar ratio	Tensile strength (kPa)
AM_{10} –Ca^{2+}	1:4	138.1 ± 3.9
AM_{10} –Ca^{2+}	1:3	149.2 ± 2.5
AM_{10} –Ca^{2+}	1:2	166.9 ± 4.1
AM_{10} –Ca^{2+}	1:1	189.3 ± 5.3
AM_{10} –Ca^{2+}	2:1	161.8 ± 3.5

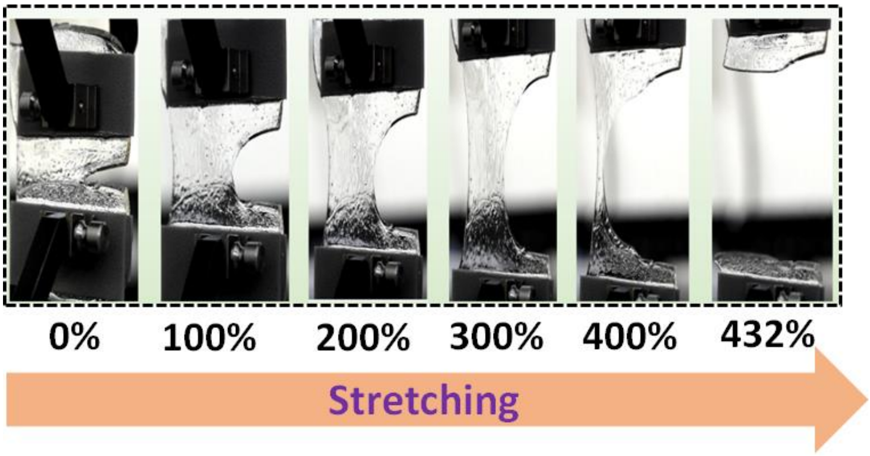

Figure S2. Photograph of crack propagation of a notched AM_{20}-Ca^{2+} Hydrogel.

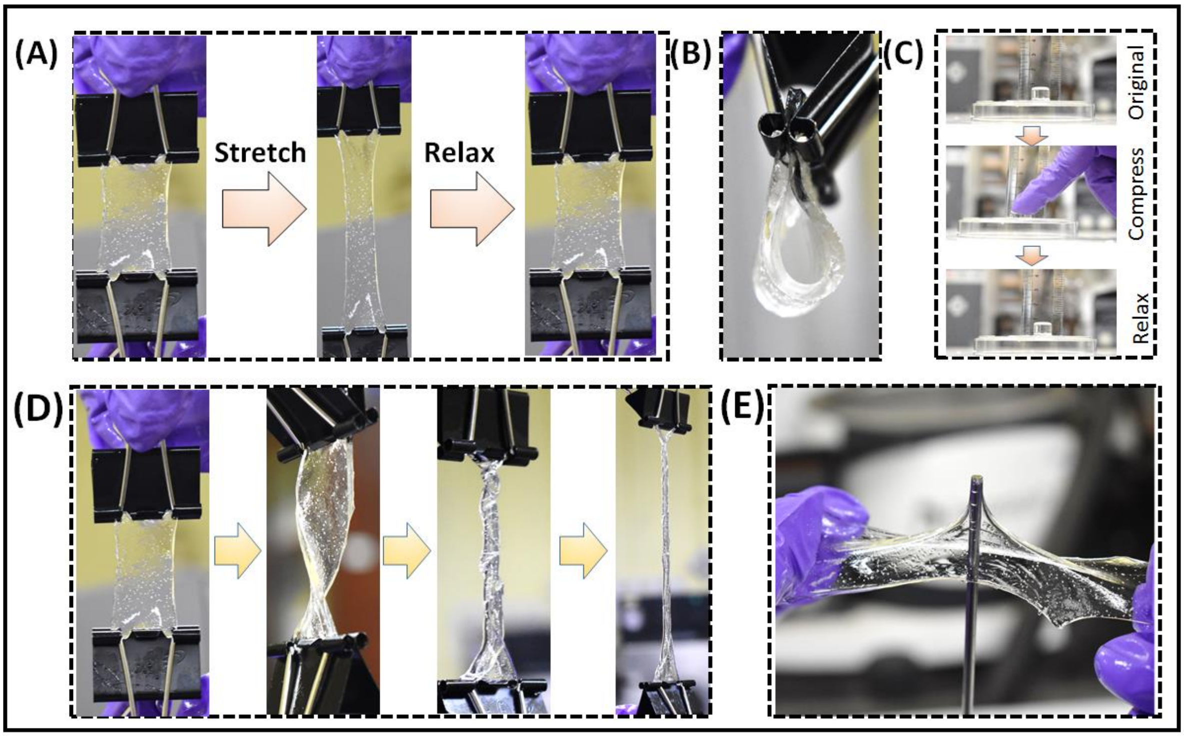

Figure S3. Photograph of hydrogel (A) During Stretching (B) Bending (C) Compression (D) Twist and stretching (E) Puncture resistance.

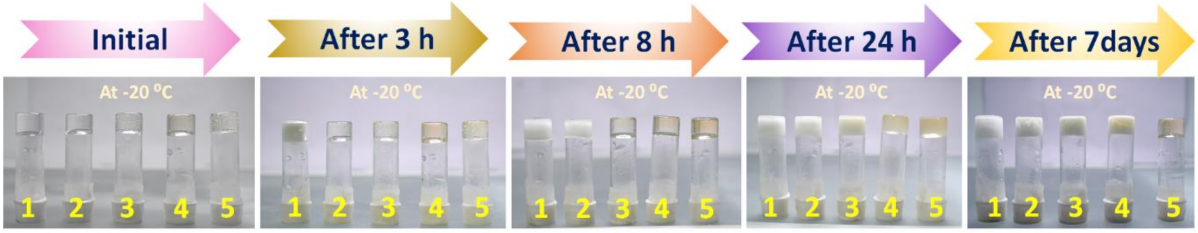

Figure S4. Variation of freezing time due the variation of hydrogel compositions: (1) AM_{10}, (2) AM_{10}-Ca^{2+}, (3) AM_{15}-Ca^{2+}, (4) AM_{20}-Ca^{2+} and (5) AM_{25}-Ca^{2+} hydrogel.

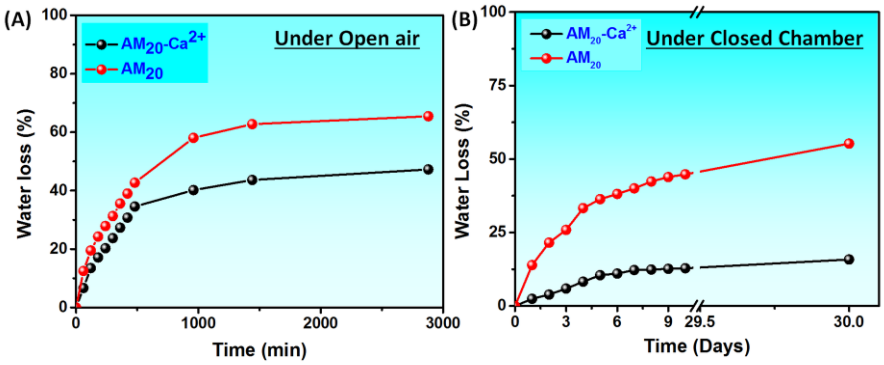

Figure S5. Water loss of AM_{20} & AM_{20}-Ca^{2+} hydrogel (A) in open air and (B) in a closed chamber (RH=75%).

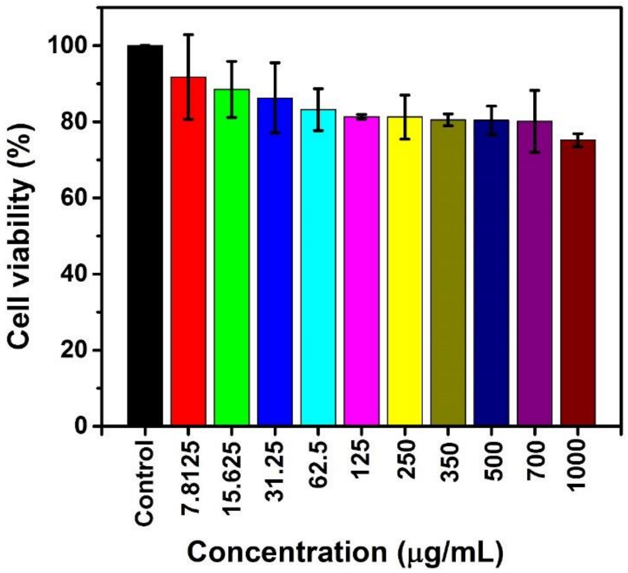

Figure S6. In-vitro cytotoxicity study (MTT assay) of AM_{20}-Ca^{2+} hydrogel. Viability of Fibroblasts L929 cells after the treatment with different concentrations of AM_{20}-Ca^{2+} Hydrogel is shown in the figure. The survivability of control cells set was assumed to be 100%. The values shown are the mean ± SD.

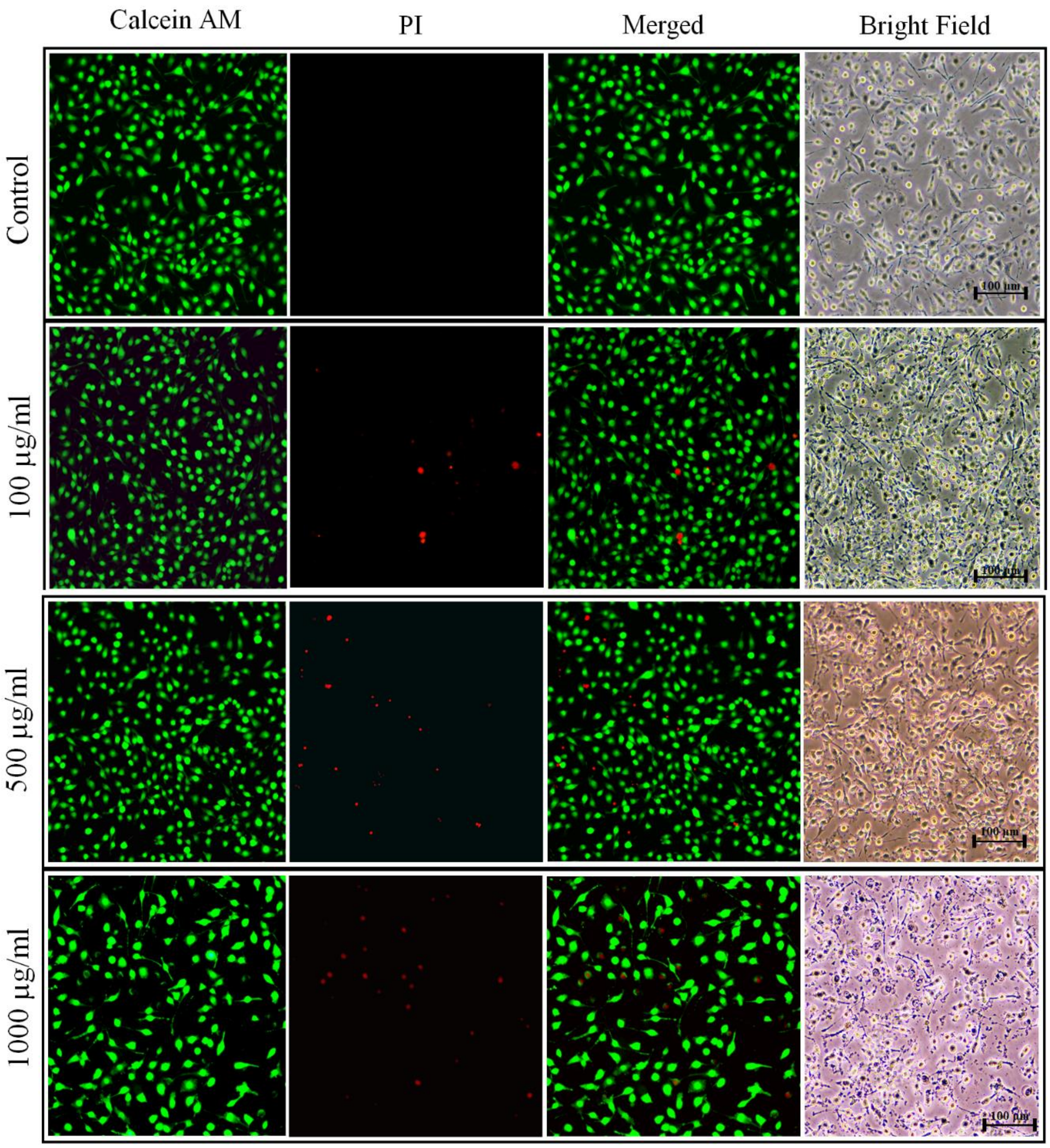

Figure S7. Live-dead cell assay images: stained with Calcein AM (green) to identify viable cells and PI (red) to identify dead ones. Cell viability did not alter or decrease at different concentrations of the hydrogel after 24 h of treatment. Magnification = 10×, Scale Bar = 100 µm. (For interpretation of the references to colour in this figure legend, the reader is referred to the Web version of this article.)

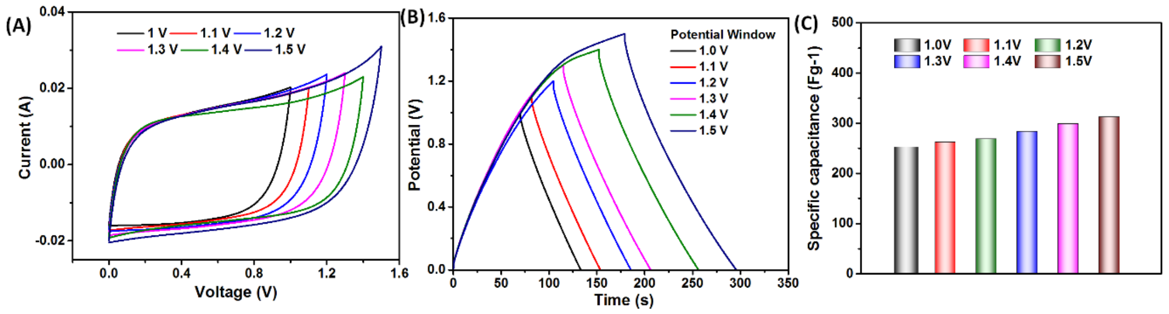

Figure S8. (A) cyclic voltammetry (CV) curve at different potential window. (B) Galvanostatic charge discharge (GCD) profile at different potential window (C) Specific capacitance at different potential window.

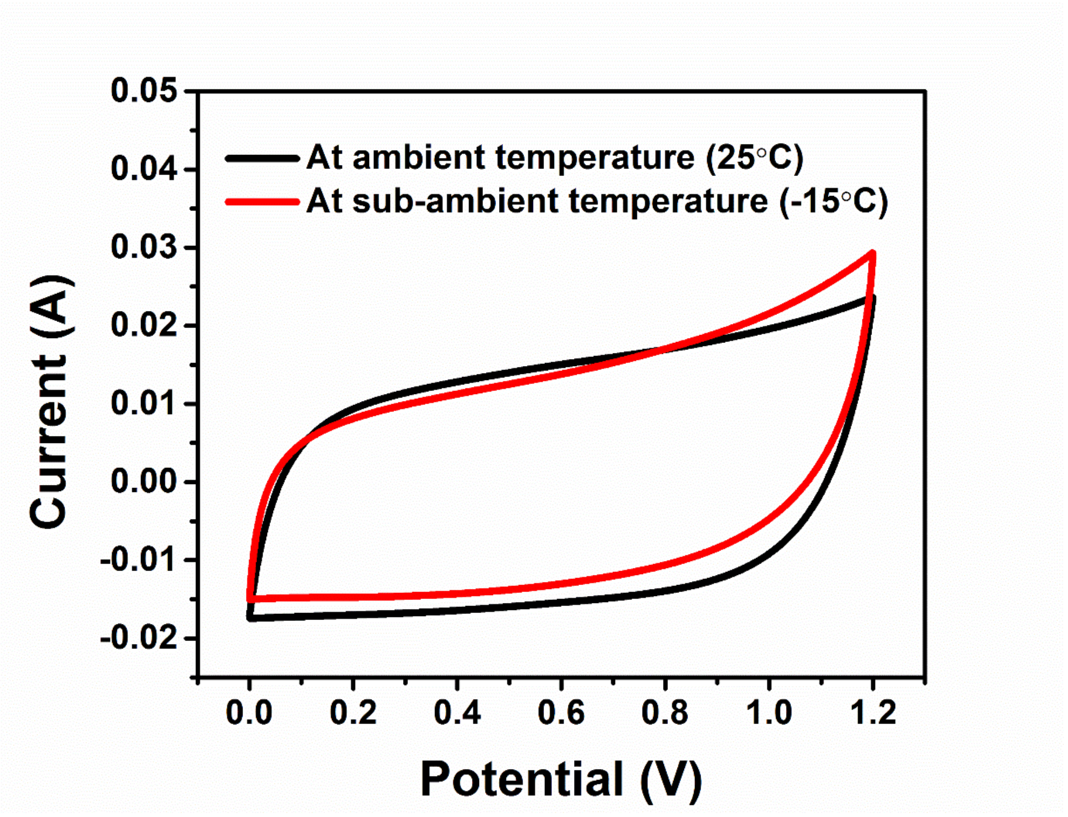

Figure S9. (A) cyclic voltammetry (CV) curve of the device at ambient temperature and subambient temperature.

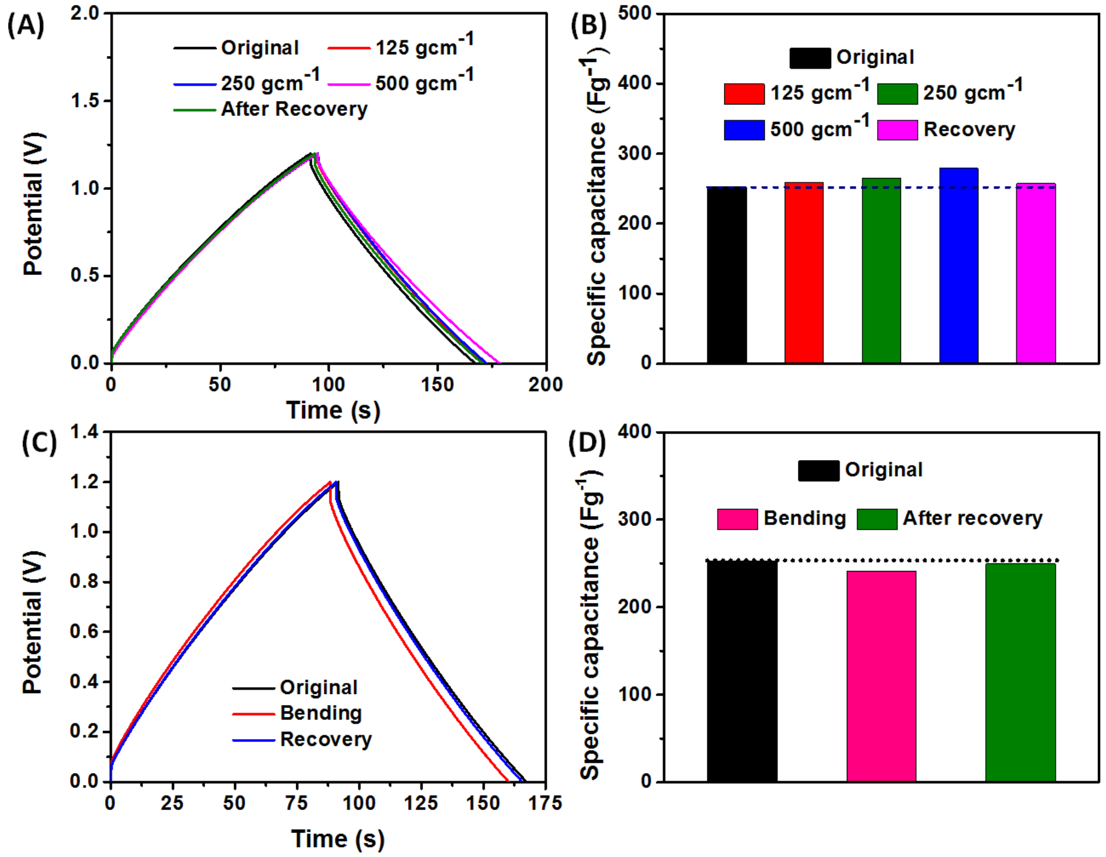

Figure S10. (A) Galvanostatic charge discharge (GCD) profile and (B) Specific capacitance after applying different load (C) Galvanostatic charge discharge (GCD) profile and (D) Specific capacitance after bending.

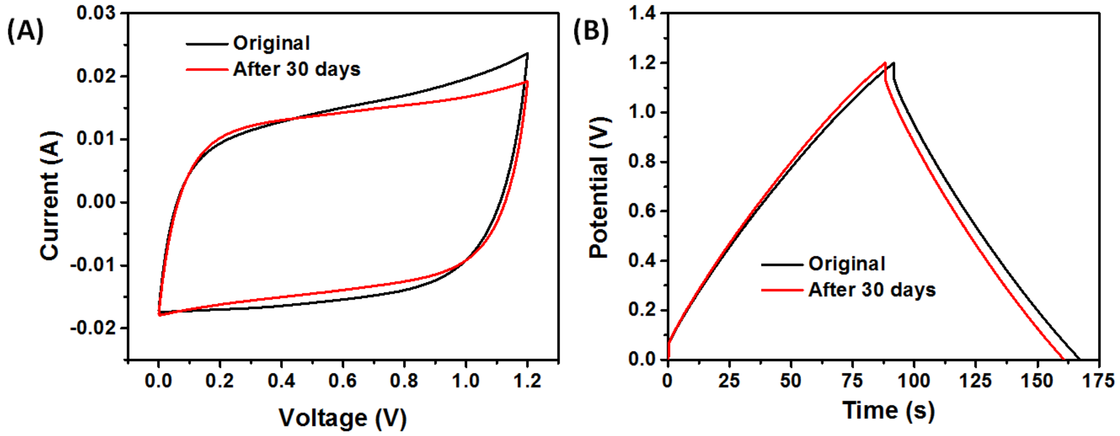

Figure S11. (A) Cyclic voltammetry (CV) curve and (B) Galvanostatic charge discharge (GCD) profile after 30 days.

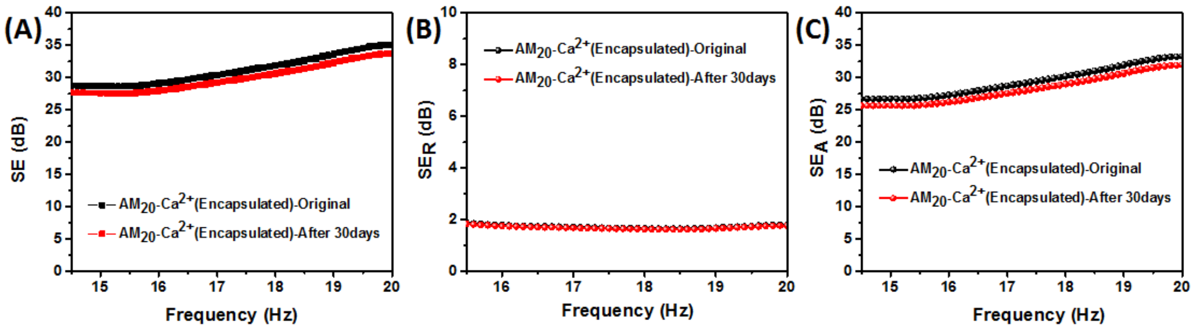

Figure S12. (A) SE, (B) SE_R, (C) SE_A of AM_{20}-Ca^{2+} hydrogel after encapsulation and after keeping 30 days of restoration.

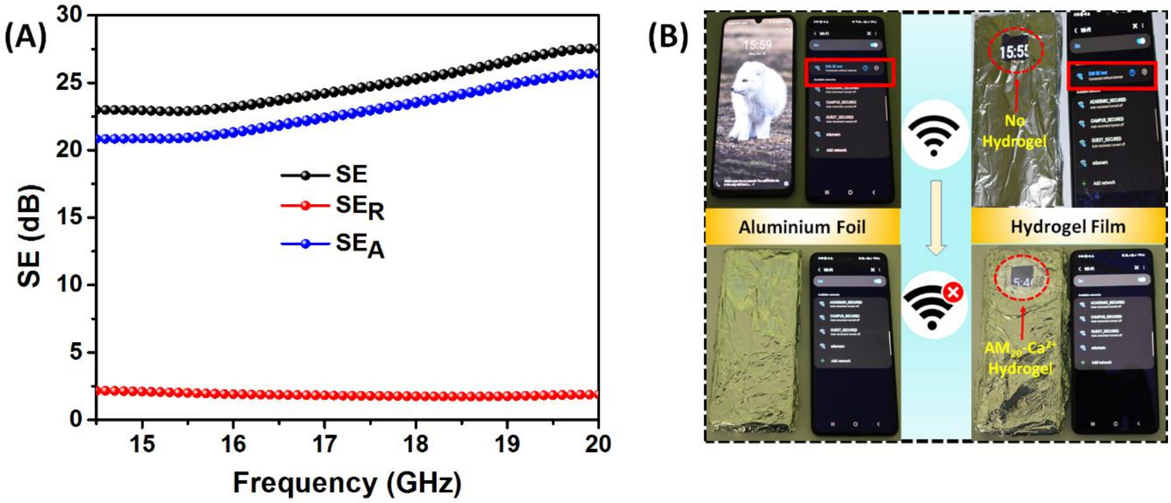

Figure S13. (A) Shielding efficiency of SE of AM_{20}-Ca^{2+} hydrogel after keeping at sub-zero temperature (-15ºC) for 12 h. (B) Real demonstration of EMI shielding capability of AM_{20}-Ca^{2+} hydrogel after blocking the device with device with this hydrogel.

Table S3. Comparison of electrochemical performance of different hydrogel electrolyte based super capacitor device.

Hydrogel	Current Density (A g-1)	Specific Capacitance (Fg-1)	Maximum Energy Density (Wh. kg^{-1})	Maximum Power Density (W kg^{-1})	Ref.
AM_{20}-Ca^{2+}	0.5	259.9	8.7	6000	This Work
	1	252.3			
	2	236.7			
	3	215			
	4	200			
	5	188.3			
	10	145			
$AM_{10}B_{10}$-Ni	0.5	173.2	8.7	6000	[5]
Li-AG/PAM	0.5	73.3	1.8	250	[6]
Li-AQ	0.5	77.4	1.7	289.4	[6]
PDA	0.5	92.7	4.6	3000	[3]
PVA-PAx	0.5	75.9	15.2	12000	[7]
CPC-CH	0.5	149.4	18.9	100	[8]
PMAI-Ni-2	0.02	98.9	-	-	[9]
PMAI-Ni-2	0.02	70	-	-	[9]
PAAK/CMC	0.3	55.1	-	-	[10]
UPyHCBA	0.5	38	1.9	1880	[11]
S-HPCD	0.5	129.8	4.2	2000	[12]
S-PVA	0.5	131	5	3272	[12]
LC-GPE	0.5	123.7	4.5	4300	[13]
DMAPS-PAA	0.5	125	4.4	2200	[14]

Table S4. Comparison of shielding efficiency of different shielding materials.

Materials	Type of materials	Frequency range (GHz)	Thickness (mm)	Total Shielding efficiency (dB)	Ref.
PAM hydrogel	Hydrogel	8.2-12.4	2 mm	15	[15]
PAM/CNF/CNT	Hydrogel	8.2-12.4	2 mm	28.5	[15]
PAM-Alginate	Hydrogel	14.5-20	2.5 mm	25.12	[16]
PAM-Alginate-Ca^{2+}	Hydrogel	14.5-20	2.5 mm	30.42	[16]
PAM-Alginate-Ca^{2+}/Ag NR	Hydrogel	14.5-20	2.5 mm	31.91-48.72	[16]
PAM-Alg-$LiCl_2$	Hydrogel	14.5-20	2.5 mm	32.98	[17]
PAM-Alg-IL	Hydrogel	14.5-20	2.5 mm	38.11	[17]
PAM-Alg-IL- LiCl	Hydrogel	14.5-20	2.5 mm	45.76	[17]
PAM/PVA6	Hydrogel	8-12	3 mm	21.6	[18]
PAM/PVA6/LiCl	Hydrogel	8.2-12.4	3 mm	35.6-37.7	[18]
PVA-PEG-SA-180/ NaCl	Hydrogel	8.2-12.4	3 mm	32.07	[19]
PVA Hydrogel	Hydrogel	8.2-12.4	2 mm	21.02	[20]
PVA-MWCNTs Hydrogel	Hydrogel	8.2-12.4	2 mm	22.34	[20]
PVA-MWCNTs(KH550)	Hydrogel	8.2-12.4	2 mm	26.69	[20]
PVA-MWCNTs(SDBS)	Hydrogel	8.2-12.4	2 mm	29.38	[20]
graphene/ PDMS	Foam	8.2-12.4	1 mm	20	[21]
PDMS/FRS	Foam	14.5-20	4 mm	24.3	[22]
PEI/graphene/Fe3O4	Foam	8.2-12.4	2.5 mm	18	[23]
GTA/PDMS	Foam	8.2-12.4	10	36.5	[24]
PEI/graphene	Foam	8.2-12.4	2.3 mm	22	[25]
graphene/polystyrene (PS)	Foam	8.2-12.4	2.5 mm	29	[26]
polymethylmethacrylate/rGO	Foam	8.2-12.4	2.4 mm	13	[27]
rGO/PU	Foam	8.2-12.4	2.5 mm	23	[28]
rGO/polyimide (PI)	Foam	8.2-12.4	0.8 mm	21	[29]
Polystyrene (PS)/CNTs	Foam	8.2-12.4	-	18.5	[30]
poly(L-lactic acid)/MWCNTs	Foam	8.2-12.4	2.54 mm	23	[31]
PP/CF	Foam	8.2-12.4	3.2 mm	25	[32]
Ag NWs/PI	Foam	8.2-12.4	5 mm	38	[33]
Ag@PPy sponge	sponge	8.2-12.4	3 mm	33	[34]
$NaIL_3$	Bulk	14.5-20	2.4 mm	50.92	[35]
$CaIL_3$	Bulk	14.5-20	2.4 mm	52.19	[35]
KIL_1	Bulk	14.5-20	2.4 mm	51	[35]
KIL_3	Bulk	14.5-20	2.4 mm	55	[35]
PVA/SWCNTs/CZTO/PDMS	Bulk	14.5-20	2.1 mm	74.81	[36]
AM_{20}	Hydrogel	14.5-20	1.5 mm	26.78	This Work
AM_{20}-Ca^{2+}	Hydrogel	14.5-20	1.5 mm	35	
AM_{20}-Ni^{2+}	Hydrogel	14.5-20	1.5 mm	39.14	
AM_{20}-Zn^{2+}	Hydrogel	14.5-20	1.5 mm	37.30	

Reference:

(1) Rivlin, R. S.; Thomas, A. G. Rupture of Rubber . I . Characteristic Energy for Tearing. *J. Polym. Sci.* **1953**, *10*, 291–318.

(2) Dai, L. xin; Zhang, W.; Sun, L.; Wang, X. huo; Jiang, W.; Zhu, Z. wen; Zhang, H. bin; Yang, C. cai; Tang, J. Highly Stretchable and Compressible Self-Healing P(AA-Co-AAm)/CoCl2 Hydrogel Electrolyte for Flexible Supercapacitors. *ChemElectroChem* **2019**, *6* (2), 467–472. https://doi.org/10.1002/celc.201801281.

(3) Dutta, A.; Panda, P.; Das, A.; Ganguly, D.; Chattopadhyay, S.; Banerji, P.; Pradhan, D.; Das, R. K. Intrinsically Freezing-Tolerant, Conductive, and Adhesive Proton Donor-Acceptor Hydrogel for Multifunctional Applications. *ACS Appl. Polym. Mater.* **2022**, *4* (10), 7710–7722. https://doi.org/10.1021/acsapm.2c01285.

(4) Xiang, C.; Li, M.; Zhi, M.; Manivannan, A.; Wu, N. A Reduced Graphene Oxide/Co3O4 Composite for Supercapacitor Electrode. *J. Power Sources* **2013**, *226*, 65–70. https://doi.org/10.1016/j.jpowsour.2012.10.064.

(5) Ghosh, A.; Pandit, S.; Kumar, S.; Ganguly, D.; Chattopadhyay, S.; Pradhan, D.; Das, R. K. Designing Dynamic Metal-Coordinated Hydrophobically Associated Mechanically Robust and Stretchable Hydrogels for Versatile, Multifunctional Applications in Strain Sensing, Actuation and Flexible Supercapacitors. *Chem. Eng. J.* **2023**, *475*, 146160. https://doi.org/10.1016/j.cej.2023.146160.

(6) Lin, T.; Shi, M.; Huang, F.; Peng, J.; Bai, Q.; Li, J.; Zhai, M. One-Pot Synthesis of a Double-Network Hydrogel Electrolyte with Extraordinarily Excellent Mechanical Properties for a Highly Compressible and Bendable Flexible Supercapacitor. *ACS Appl. Mater. Interfaces* **2018**, *10* (35), 29684–29693. https://doi.org/10.1021/acsami.8b11377.

(7) Zhao, J.; Lu, Y.; Liu, Y.; Liu, L.; Yin, J.; Sun, B.; Wang, G.; Zhang, Y. A Self-Healing PVA-Linked Phytic Acid Hydrogel-Based Electrolyte for High-Performance Flexible Supercapacitors. *Nanomaterials* **2023**, *13* (3), 380. https://doi.org/10.3390/nano13030380.

(8) Peng, K.; Zhang, J.; Yang, J.; Lin, L.; Gan, Q.; Yang, Z.; Chen, Y.; Feng, C. Green Conductive Hydrogel Electrolyte with Self-Healing Ability and Temperature Adaptability for Flexible Supercapacitors. *ACS Appl. Mater. Interfaces* **2022**, *14*, 39404. https://doi.org/10.1021/acsami.2c11973.

(9) Dutta, A.; Ghosal, K.; Sarkar, K.; Pradhan, D.; Das, R. K. From Ultrastiff to Soft Materials : Exploiting Dynamic Metal – Ligand Cross-Links to Access Polymer Hydrogels Combining Customized Mechanical Performance and Tailorable Functions by Controlling Hydrogel Mechanics. *Chem. Eng. J.* **2021**, *419*, 129528. https://doi.org/10.1016/j.cej.2021.129528.

(10) Deng, Y.; Wang, H.; Zhang, K.; Shao, J.; Qiu, J.; Wu, J.; Wu, Y.; Yan, L. A High-Voltage Quasi-Solid-State Flexible Supercapacitor with a Wide Operational Temperature Range Based on a Low-Cost “Water-in-Salt” Hydrogel Electrolyte. *Nanoscale* **2021**, *13* (5), 3010–3018. https://doi.org/10.1039/d0nr08437a.

(11) Zhao, Y.; Liang, Q.; Mugo, S. M.; An, L.; Zhang, Q.; Lu, Y. Self-Healing and Shape-Editable Wearable Supercapacitors Based on Highly Stretchable Hydrogel Electrolytes. *Adv. Sci.* **2022**, *9* (24), 2201039. https://doi.org/10.1002/advs.202201039.

(12) Xu, S.; Liang, X.; Ge, K.; Yuan, H.; Liu, G. Supramolecular Gel Electrolyte-Based Supercapacitors with a Comparable Dependence of Electrochemical Performances on Electrode Thickness to Those Based on Bulk Electrolyte Solutions. *ACS Appl. Energy Mater.* **2022**, *5* (3), 2929–2936. https://doi.org/10.1021/acsaem.1c03607.

(13) Qiu, F.; Huang, Y.; Hu, X.; Li, B.; Zhang, X.; Luo, C.; Li, X.; Wang, M.; Wu, Y.; Cao, H. An Ecofriendly Gel Polymer Electrolyte Based on Natural Lignocellulose with Ultrahigh Electrolyte Uptake and Excellent Ionic Conductivity for Alkaline Supercapacitors. *ACS Appl. Energy Mater.* **2019**, *2* (8), 6031–6042. https://doi.org/10.1021/acsaem.9b01150.

(14) Feng, E.; Gao, W.; Li, J.; Wei, J.; Yang, Q.; Li, Z.; Ma, X.; Zhang, T.; Yang, Z. Stretchable, Healable, Adhesive, and Redox-Active Multifunctional Supramolecular Hydrogel-Based Flexible Supercapacitor. *ACS Sustain. Chem. Eng.* **2020**, *8* (8), 3311–3320. https://doi.org/10.1021/acssuschemeng.9b07153.

(15) Yang, W.; Shao, B.; Liu, T.; Zhang, Y.; Huang, R.; Chen, F.; Fu, Q. Robust and Mechanically and Electrically Self-Healing Hydrogel for Efficient Electromagnetic Interference Shielding. *ACS Appl. Mater. Interfaces* **2018**, *10* (9), 8245–8257. https://doi.org/10.1021/acsami.7b18700.

(16) De, A.; Paria, S.; Bera, A.; Si, S. K.; Bera, S.; Khatua, B. B. Elastomer Encapsulated Silver Nanorod Dispersed Polyacrylamide-Alginate Hydrogel for Water-Pressure-Dependent EMI Shielding Application. *ACS Appl. Eng. Mater.* **2023**, *1* (1), 95–108. https://doi.org/10.1021/acsaenm.2c00020.

(17) De, A.; Singh, P. P.; Mondal, A.; Khatua, B. B. Lithium Chloride-Driven Enhanced Conductivity of Silicone-Encapsulated Polyacrylamide/Alginate/Ionic Liquid-Based Transparent Hydrogel for High-Performance Pressure-Sensitive EMI Shielding Applications. *J. Mater. Sci.* **2023**, *58* (40), 15917–15932. https://doi.org/10.1007/s10853-023-09019-9.

(18) Yuan, S.; Dai, T.; Jiang, X.; Zou, H.; Liu, P. Transparent and Environmentally Adaptive Semi-Interpenetrating Network Hydrogels for Electromagnetic Interference Shielding. *ACS Appl. Polym. Mater.* **2023**. https://doi.org/10.1021/acsapm.3c01381.

(19) Xu, Y.; Pei, M.; Du, J.; Yang, R.; Pan, Y.; Zhang, D.; Qin, S. A Tough, Anticorrosive Hydrogel Consisting of Bio-Friendly Resources for Conductive and Electromagnetic Shielding Materials. *New J. Chem.* **2023**, *47* (29), 13721–13728. https://doi.org/10.1039/d3nj02339g.

(20) Xu, Y.; Tan, Y.; Xue, Y.; Pei, M.; Zhang, D.; Liu, S.; Qin, S. A Novel PVA/MWCNTs Hydrogel with High Toughness and Electromagnetic Shielding Properties. *Polym. Eng. Sci.* **2023**, *63* (11), 3555–3564. https://doi.org/10.1002/pen.26465.

(21) Chen, Z.; Xu, C.; Ma, C.; Ren, W.; Cheng, H. M. Lightweight and Flexible Graphene Foam Composites for High-Performance Electromagnetic Interference Shielding. *Adv. Mater.* **2013**, *25* (9), 1296–1300. https://doi.org/10.1002/adma.201204196.

(22) Wang, S.; Li, D.; Meng, W.; Jiang, L.; Fang, D. Scalable, Superelastic, and Superhydrophobic MXene/Silver Nanowire/Melamine Hybrid Sponges for High-Performance Electromagnetic Interference Shielding. *J. Mater. Chem. C* **2022**, *10* (13), 5336–5344. https://doi.org/10.1039/d2tc00516f.

(23) Shen, B.; Zhai, W.; Tao, M.; Ling, J.; Zheng, W. Lightweight, Multifunctional

Polyetherimide/Graphene@Fe3O 4 Composite Foams for Shielding of Electromagnetic Pollution. *ACS Appl. Mater. Interfaces* **2013**, *5* (21), 11383–11391. https://doi.org/10.1021/am4036527.

(24) Chen, J.; Shen, B.; Jia, X.; Liu, Y.; Zheng, W. Lightweight and Compressible Anisotropic Honeycomb-like Graphene Composites for Highly Tunable Electromagnetic Shielding with Multiple Functions. *Mater. Today Phys.* **2022**, *24* (April), 100695. https://doi.org/10.1016/j.mtphys.2022.100695.

(25) Ling, J.; Zhai, W.; Feng, W.; Shen, B.; Zhang, J.; Zheng, W. G. Facile Preparation of Lightweight Microcellular Polyetherimide/Graphene Composite Foams for Electromagnetic Interference Shielding. *ACS Appl. Mater. Interfaces* **2013**, *5* (7), 2677–2684. https://doi.org/10.1021/am303289m.

(26) Yan, D. X.; Ren, P. G.; Pang, H.; Fu, Q.; Yang, M. B.; Li, Z. M. Efficient Electromagnetic Interference Shielding of Lightweight Graphene/Polystyrene Composite. *J. Mater. Chem.* **2012**, *22* (36), 18772–18774. https://doi.org/10.1039/c2jm32692b.

(27) Zhang, H. Bin; Yan, Q.; Zheng, W. G.; He, Z.; Yu, Z. Z. Tough Graphene-Polymer Microcellular Foams for Electromagnetic Interference Shielding. *ACS Appl. Mater. Interfaces* **2011**, *3* (3), 918–924. https://doi.org/10.1021/am200021v.

(28) Gavgani, J. N.; Adelnia, H.; Zaarei, D.; Moazzami Gudarzi, M. Lightweight Flexible Polyurethane/Reduced Ultralarge Graphene Oxide Composite Foams for Electromagnetic Interference Shielding. *RSC Adv.* **2016**, *6* (33), 27517–27527. https://doi.org/10.1039/c5ra25374h.

(29) Li, Y.; Pei, X.; Shen, B.; Zhai, W.; Zhang, L.; Zheng, W. Polyimide/Graphene Composite Foam Sheets with Ultrahigh Thermostability for Electromagnetic Interference Shielding. *RSC Adv.* **2015**, *5* (31), 24342–24351. https://doi.org/10.1039/c4ra16421k.

(30) Yang, Y.; Gupta, M. C.; Dudley, K. L.; Lawrence, R. W. Novel Carbon Nanotube - Polystyrene Foam Composites for Electromagnetic Interference Shielding. *Nano Lett.* **2005**, *5* (11), 2131–2134. https://doi.org/10.1021/nl051375r.

(31) Kuang, T.; Chang, L.; Chen, F.; Sheng, Y.; Fu, D.; Peng, X. Facile Preparation of Lightweight High-Strength Biodegradable Polymer/Multi-Walled Carbon Nanotubes Nanocomposite Foams for Electromagnetic Interference Shielding. *Carbon N. Y.* **2016**, *105*, 305–313. https://doi.org/10.1016/j.carbon.2016.04.052.

(32) Ameli, A.; Jung, P. U.; Park, C. B. Electrical Properties and Electromagnetic Interference Shielding Effectiveness of Polypropylene/Carbon Fiber Composite Foams. *Carbon N. Y.* **2013**, *60*, 379–391. https://doi.org/10.1016/j.carbon.2013.04.050.

(33) Ma, J.; Wang, K.; Zhan, M. A Comparative Study of Structure and Electromagnetic Interference Shielding Performance for Silver Nanostructure Hybrid Polyimide Foams. *RSC Adv.* **2015**, *5* (80), 65283–65296. https://doi.org/10.1039/c5ra09507g.

(34) Yu, L.; Yang, Q.; Liao, J.; Zhu, Y.; Li, X.; Yang, W.; Fu, Y. A Novel 3D Silver Nanowires@polypyrrole Sponge Loaded with Water Giving Excellent Microwave Absorption Properties. *Chem. Eng. J.* **2018**, *352* (July), 490–500. https://doi.org/10.1016/j.cej.2018.07.047.

(35) Singh, P. P.; Mondal, A.; Maity, P.; Khatua, B. B. Ionic Liquid-Dispersed PDMS/SWCNT/Ag@Ni Hybrid Composite for Temperature- and Pressure-Sensitive Smart Electromagnetic Shielding Applications. *J. Mater. Chem. C* **2023**, *11* (31), 10584–10597. https://doi.org/10.1039/d3tc01416a.

(36) Singh, P. P.; De, A.; Khatua, B. B. Hydro-Tunable CZTO/SWCNT/PVA/PDMS Hybrid Composites for Smart Green EMI Shielding. *J. Mater. Chem. C* **2022**, *10* (44), 16903–16913. https://doi.org/10.1039/d2tc03762a.
